# Cardiogenic Necrotizing Enterocolitis in Infants with Congenital Heart Disease: A Systematic Review and Meta-analysis

**DOI:** 10.1007/s00246-024-03686-4

**Published:** 2024-10-29

**Authors:** Ivor B. Asztalos, Stephanie N. Hill, Dustin B. Nash, Susan K. Schachtner, Kelsey J. Palm

**Affiliations:** 1 Division of Cardiology, Department of Pediatrics, Children’s Hospital of Philadelphia and University of Pennsylvania Perelman School of Medicine, 3401 Civic Center Blvd, Philadelphia, PA 19104 USA; 2 Division of Cardiology, Department of Pediatrics, Children’s Hospital Colorado, University of Colorado Denver, 13123 E 16th Ave, Anschutz Medical Campus, Aurora, CO 80045-2560 USA; 3 Clinical Nutrition, Cardiac Center, Children’s Hospital of Philadelphia, 3401 Civic Center Blvd, Philadelphia, PA 19104 USA; 4 Division of Cardiology, Department of Pediatrics, Children’s Hospital of Philadelphia and University of Pennsylvania Perelman School of Medicine, 3401 Civic Center Blvd, 8NW85, Philadelphia, PA 19104 USA

**Keywords:** Necrotizing enterocolitis, Congenital cardiac disease, Incidence, Risk factors, Systematic review, Meta-analysis

## Abstract

Necrotizing enterocolitis (NEC) is a known cause of morbidity and mortality in infants with congenital heart disease (CHD), but reports about the burden of cardiogenic NEC frequently conflict. To synthesize the extant literature on the incidence, risk factors, and prognosis of NEC in patients with CHD. Medline, Cochrane, and EMBASE were searched from 1946 through 2023 for studies of NEC in infants 0–12 months of age with CHD. Risk of bias was assessed with validated tools for incidence and risk factors. Pooled estimates were meta-analyzed by risk of bias or synthesized without meta-analysis. Eighty-six studies with a total of 67,924 participants were included. The incidence of cardiogenic NEC was 7.1% (95% CI 4.7–10.5%) in term infants and 13.0% (10.2–16.5%) in low birthweight preterm infants. NEC required surgery in 0.8% (0.5–1.1%) of term and 2.7% (2.0–3.7%) of premature infants, respectively. Only gestational age and birth weight were consistently associated with risk of NEC. Restricting pooled estimates to studies of moderate or low risk of bias significantly reduced the number of studies included. Necrotizing enterocolitis is a common cause of morbidity in infants with CHD, but additional research is needed to determine which infants are at highest risk of developing NEC and would benefit most from a change in management. This systematic review and meta-analysis was conducted according to a prespecified protocol registered at the Prospective Register of Systematic Reviews (CRD42021282114).

**Supplementary Information:**

The online version contains supplementary material available at 10.1007/s00246-024-03686-4.

## Introduction

Necrotizing enterocolitis (NEC) is a severe disease of the gastrointestinal tract characterized by inflammation, ischemia, and necrosis of the bowel, associated with increased morbidity and mortality in infants. While NEC classically occurs in premature infants, congenital heart disease (CHD) has long been recognized as a risk factor for NEC in both term and premature infants. There is growing consensus that cardiogenic NEC (cNEC) is a distinct clinical entity from NEC of prematurity highlighting the need to quantify the burden of NEC in this specific population [1].

Although an extensive literature describes the incidence of, risk factors for, and prognosis of NEC in CHD patients, there is significant inconsistency in the estimates of these outcomes. Fundamental background knowledge, such as the proportion of term infants with CHD who develop NEC, have yet to be definitively answered. Estimates of the incidence of cardiogenic NEC range widely, from less than 1% to over 60%, and can differ by more than a factor of ten within the same population, limiting our ability to quantify the impact of this potentially devastating complication [2–4]. Conflicting evidence for which specific cardiac anomalies or other risk factors increase the risk of developing NEC further limits clinicians’ ability to manage this important outcome.

To date a small number of systematic reviews have shed some light on this disease process but are limited in scope and focus primarily on the impact of feeding protocols [5–9]. Given the additional morbidity and mortality conferred by developing NEC in this already fragile population, we performed a systematic review and meta-analysis to synthesize the relevant literature to determine the incidence of, risk factors for, and prognosis of cardiogenic NEC in infants with CHD.

## Methods

This systematic review and meta-analysis was conducted according to a prespecified protocol registered at the Prospective Register of Systematic Reviews (CRD42021282114) and is reported in accordance with the Preferred Reporting Items for Systematic Reviews and Meta-Analyses (PRISMA) 2020 guidelines [10].

### Search Strategy

In concert with a University of Pennsylvania librarian we [author IBA] systematically searched Medline, Cochrane, and EMBASE, including its gray literature repository of conference abstracts, from 1946 (inception) through 2023. The search strategy included both medical subject headings (MeSH) and key terms and is fully delineated in Supplemental Method 1. While no filters were used at the search stage, only reports in English, of human patients, and of infants were included. Additionally, we reviewed for eligibility any reports identified in the reference lists of both included reports and pertinent review articles.

### Eligibility Criteria

Any study which reported on either the incidence, risk factors, or prognosis of NEC in infants 0–12 months of age with structural CHD were eligible for inclusion. As the existing literature on NEC of prematurity is robust and explicitly not the focus of this review, studies or subgroups of participants whose only structural heart defect was an isolated patent ductus arteriosus (PDA) or patent foramen ovale (PFO) were excluded. Studies of systematic case series (e.g., sequential inclusion of all patients who underwent cardiac surgery) were eligible, but convenience sample case series and case reports were excluded. In rare cases, when the same or overlapping cohorts of participants from the same study were included in multiple reports, only the report with the largest sample size was retained.

Records identified from the database searches and the reference lists of included studies were imported into a reference management system (Rayyan) and duplicates removed [11]. Each abstract was reviewed independently for inclusion by two of three investigators [authors IBA, KJP, and SNH] and all conflicts resolved by the entire group’s consensus at a later review. All reports were then obtained, and the full manuscripts reviewed for inclusion and data abstraction.

### Data Collection

One of two reviewers [authors IBA, KJP] extracted the primary data from each of the included studies using a standardized data collection form. Owing to the volume of included studies and the length of time since publication of the earliest reports, study authors were not contacted for missing data or for clarification of methods. For each study we collected the overall study design, how the NEC outcome (either all and/or surgical) was defined, inclusion/exclusion criteria for the overall study and individual subgroups, investigated risk factors, and potential confounding covariates. Where possible, descriptive statistics or participant level data were used to calculate unreported estimates of interest, but primary missing data were not requested from corresponding authors.

### Assessment of Risk of Bias

One of two investigators [authors IBA, KJP] assessed the risk of bias (RoB) in each included study. Two risk of bias assessment tools were used as the relevance of specific study elements and limitations varies depending on the study design and research question (e.g., incidence vs risk factor). For studies evaluating the incidence and prognosis of cNEC, the Joanna Briggs Institute Critical Appraisal Checklist for Prevalence Studies was employed, and the Newcastle–Ottawa Quality Assessment Scale was used to assess risk of bias for both case–control and cohort studies investigating risk factors for cNEC (Supplemental Methods 2–3) [12, 13].

### Outcomes

The primary outcome was the incidence of NEC among infants with structural congenital heart disease. Secondary outcomes included: incidence of surgical NEC, modifiable and non-modifiable risk factors for development of NEC, and hospital length of stay.

If ascertainable from the reported data, NEC was defined as modified Bell’s Stage IIA or greater. Otherwise, it was defined according to the methodology outlined in the study. Surgical NEC was defined as the need for laparotomy with bowel resection, peritoneal drain, or ostomy placement. Given the substantial work that has already been performed summarizing the evidence for feeding protocols, we did not consider this particular risk factor in our analyses [5–9].

### Subgroup Analyses

Studies were stratified across three dimensions for meta-analysis: (1) all CHD vs. specific lesions, (2) all or term infants vs. premature infants, and (3) risk of bias. Studies reported outcomes either for a specific anatomical subgroup exclusively (e.g., single ventricle patients) or encompassed all CHD patients with or without reporting on individual subgroups. Cohorts containing a diverse group of cardiac lesions, e.g., any congenital heart disease or requiring cardiac surgery by two months of age, were pooled for the all-CHD meta-analysis. Conversely, studies addressing outcomes in specific anatomical subgroups exclusively were omitted from this analysis, as even in aggregate, they represent a distinct population of CHD. However, data from these studies were merged with subgroup data from the all-encompassing CHD studies on a lesion-by-lesion basis for meta-analyses of specific anatomical subtypes. Nearly 40% (30/77) of all anatomical subgroups or dedicated studies focused on single ventricle patients. We further categorized these studies into the following: (1) All single ventricle patents irrespective of palliation type, (2) Surgical Stage I patients exclusively, and (3) Hybrid stage I patients exclusively.

As prematurity/low birth weight are known risk factors for the development of NEC, studies which reported exclusively on these populations were analyzed separately. Not all combinations of strata were present, e.g., there were no moderate or low risk of bias studies of patients with transposition of the great arteries.

### Data Synthesis

Incidence of outcomes for individual studies were calculated from the extracted study level data and 95% confidence intervals calculated using the Wilson score without continuity correction [14]. Given significant heterogeneity in methodology and sample size between studies, a Hartung–Knapp random-effects generalized linear mixed model with logit transformation was fit to calculate pooled incidence rates [15]. Length of stay medians were analyzed using the median of the difference of medians method [16]. Overall and within-subgroup heterogeneity was assessed quantitatively by calculating the *I*^2^, *τ*^2^ (in logit units), and prediction intervals, while qualitative heterogeneity was assessed based on study methodology. The relationship between the logarithm of cohort size and incidence was assessed with simple linear regression. All analyses were performed in R (R Core Team, Vienna, Austria) using the meta and metamedian packages [author IBA] [17, 18].

For studies which reported outcomes for multiple subgroups (e.g., cNEC incidence for hypoplastic left heart syndrome, isolated coarctations, etc.) each subgroup was considered an independent study and treated as the unit of analysis. For studies which reported nested subgroups (e.g., all single ventricles and all single ventricles who underwent surgical Stage I), non-overlapping groups within the same study were created at the authors’ discretion to preserve the unit of analysis for clarity and clinical relevance.

Due to significant heterogeneity in study methodology and universally high risk of bias, data on risk factors for cNEC were not amenable for meta-analysis. Therefore, results are reported per Synthesis Without Meta-analysis (SWiM) guidelines. Although odds ratios could be extracted or calculated, the standardization method was reduced to whether an association with cNEC (*p* < 0.05) was found in multivariable analysis. Results from studies which made no attempt to adjust for any confounders were excluded from this data synthesis, however, which potential confounders were included did not determine eligibility. The extracted data from the studies used for analysis and the analytical code are available upon request from the corresponding author.

## Results

**Fig. 1:**
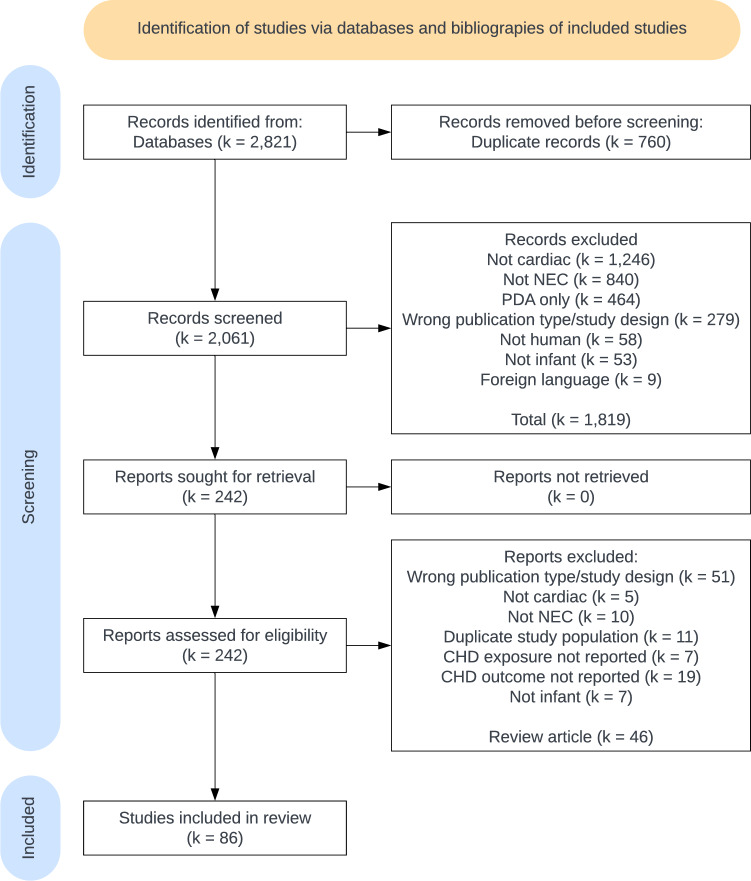
Flow diagram of search results, excluded reports, and included studies. Studies may meet more than one exclusion criteria

From the three databases and the references of included studies, 2821 records were identified, 2061 abstracts screened, and 242 full manuscripts and abstracts reviewed (Fig. 1). Eighty-six studies were included in the final review with a total of 67,924 participants [2–4, 19–100]. Study characteristics are listed in Supplemental Table 1. For studies of incidence of cNEC and sNEC, results from the risk of bias assessments are provided in Supplemental Tables 2–4.

### Cardiogenic NEC Incidence

**Fig. 2:**
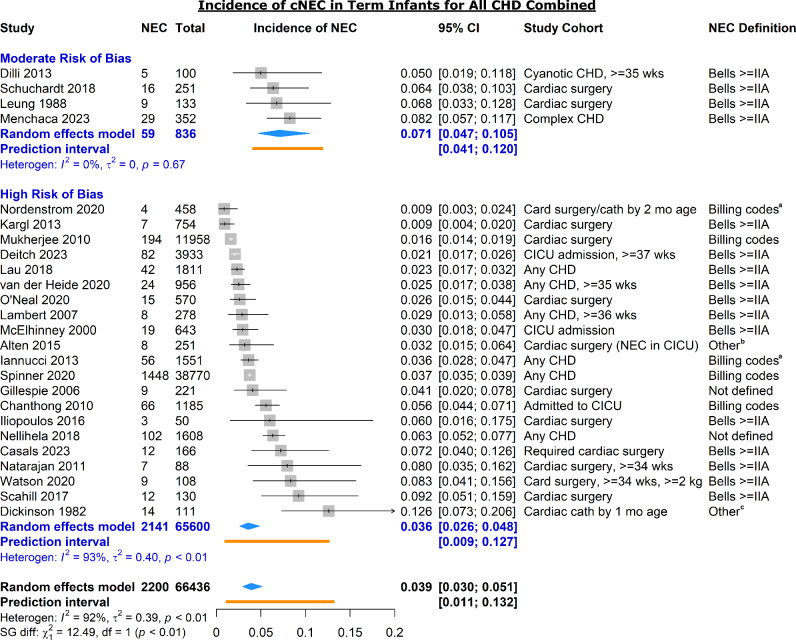
Forest plot of incidence of cardiogenic NEC in all or term infants by study risk of bias. *RoB* risk of bias; *CICU* cardiac intensive care unit; *CHD* congenital heart disease. Complex CHD excludes isolated atrial and ventricular septal defects. ^a^Internal cardiac surgical complications registry; ^b^Clinical and radiographic findings requiring antibiotics or laparotomy; ^c^Hematochezia and abdominal distension

A total of 25 studies reported on the incidence of cardiogenic NEC among all infants with all forms of CHD combined (excluding isolated PDA and PFO, Fig. 2) [2, 4, 19, 20, 25, 28, 30, 34, 38, 40, 43, 44, 55, 57, 64, 65, 69, 72, 74, 75, 87, 88, 90, 93, 97]. These studies were either restricted to term infants or included both term and preterm infants but did not report incidences stratified by gestational age. The pooled incidence of cNEC of the studies assessed as moderate risk of bias (none were low) was 7.1% (95% CI 4.7–10.5%) [20, 40, 64, 90]. The range of individual incidences of these studies was narrow (5.0–8.2%), and subsequently no heterogeneity was identified in this subgroup (*p* = 0.67, PI 4.1–12.0%).

The incidence of cNEC in the remaining twenty-one studies adjudicated as high risk of bias was lower at 3.6% (95% CI 2.6–4.8%). Both qualitative and quantitative assessments of heterogeneity were high in this subgroup (PI 0.9–12.7%, *I*^2^ = 93%). Studies with larger sample sizes were more likely to use administrative databases to identify cNEC cases and to report lower incidences (Supplemental Fig. 1). Indeed, the larger the cohort size, the lower the reported incidence of cNEC. For every tenfold increase in sample size the incidence of NEC decreased by 2.6 per 100 infants (*p* < 0.001).

**Fig. 3:**
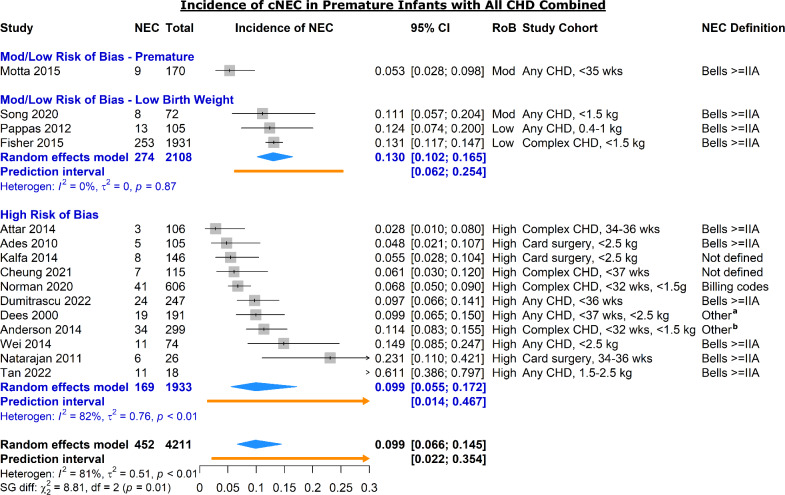
Forest plot of incidence of cardiogenic NEC in preterm or low birth weight infants by study risk of bias and low birth weight criteria. *RoB* risk of bias; *CHD* congenital heart disease. Complex CHD excludes isolated atrial and ventricular septal defects. ^a^Each of: clinical signs, x-ray findings, and required antibiotics or NPO; ^b^Clinical findings requiring antibiotics or abdominal surgery

A total of 15 studies reported on the incidence of cNEC among premature or low birth weight infants with all forms of CHD (Fig. 3) [3, 26, 35, 38, 39, 48, 50–52, 54, 56, 73, 76, 78, 86]. Only four studies were considered moderate or low risk of bias. Of these, three used an inclusion criterion of low birth weight [39, 54, 76], and one used a criterion of based only on gestational age [56].

In the three low or moderate risk of bias studies in infants of either very low or extremely low birth weight (VLBW or ELBW), the pooled incidence of cNEC was 13.0% (95% CI 10.2–16.5%) [39, 54, 76]. No heterogeneity was observed (*I*^2^ = 0%). The one moderate risk of bias study of premature infants without a birth weight restriction reported a lower incidence of 5.3% (CI 95% 2.8–9.9%) [56]. The average birth weight in this study was 1457 g—at or above the weight-based cutoffs of the three weight-based studies. The remaining eleven studies were all high risk of bias and reported a lower pooled incidence of 9.9% (CI 5.5–17.2%). Owing to differences in how cNEC cases were identified and staged, heterogeneity in the subgroup of high risk of bias studies was substantial (*I*^2^ = 82%, *τ*^2^ = 0.76, PI 1.4–46.7%).

Of the 30 studies which reported cNEC incidence in single ventricle patients, only two studies were adjudicated as moderate risk of bias and none were low (Supplemental Fig. 2). In a cohort of single ventricle patients, 81% of whom underwent a Norwood procedure and 1% of whom had a hybrid stage I procedure, Blanco et al. reported an incidence of cNEC of 4.7% (2.0–10.5%) [92]. In hybrid stage I patients exclusively, Carpenito et al. reported a cNEC incidence of 8.8% (5.0–15.1%) [58]. Incidence of cNEC in the 28 high risk of bias studies was unreliable, ranging widely from 0 to 27%. Even after stratifying by Stage I type (surgical, hybrid, combined) heterogeneity remained substantial, e.g., the prediction interval ranged from less than 1% to more than 72% for the exclusive hybrid stage I studies [37, 42, 49, 59, 62, 82].

Incidences of cNEC in other anatomical subgroups in term or syndromic patients are summarized in Supplemental Fig. 3. There was high variability in how cNEC and the subgroups were defined, and the risk of bias was high in 44 of the 47 studies. Estimates of the incidence of cNEC in each study ranged from as low as 0% to as high as 50%, albeit in small cohorts. Notably, the pooled incidence of cNEC in ductal-dependent lesions was only 4% (2–7%). Five studies reported on the incidence of cNEC in premature or LBW infants with specific cardiac anomalies (Supplemental Fig. 4) [26, 46, 54, 56, 95]. Incidence of cNEC in specific cardiac lesions investigated by the two studies of moderate or low risk of bias ranged from a low of 3.8% in mild CHD patients to a high of 18% in patients with tricuspid or pulmonary atresia [54, 56].

### Surgical Cardiogenic NEC Incidence

**Fig. 4:**
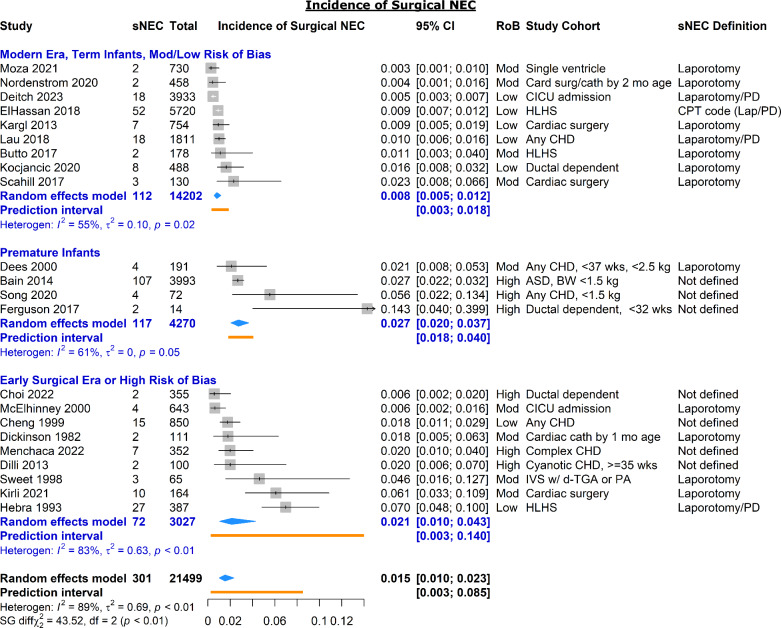
Forest plot of surgical NEC (sNEC) in infants with CHD stratified by surgical era, prematurity, and study risk of bias. *RoB* risk of bias; *PD* peritoneal drain; *HLHS* hypoplastic left heart syndrome; *ASD* atrial septal defect; *IVS* intact ventricular septum; *TGA* transposition of the great arteries; *PA* pulmonary atresia

Twenty-two studies reported on the incidence of surgical NEC among infants with CHD (Fig. 4). These studies were divided post hoc into three categories to limit heterogeneity within subgroups: (1) moderate/low risk of bias studies from the modern era [2, 4, 44, 62, 65, 68, 79, 88, 94], (2) studies in premature infants [26, 46, 76, 95], and (3) studies from an early surgical era (prior to 2000) or with high risk of bias [19, 21, 23–25, 40, 80, 84, 90]. Studies from the last group were considered unreliable estimates of the risk of surgical NEC in the modern era.

In moderate/low risk of bias studies from the modern era the pooled incidence of sNEC was 0.8% (95% CI 0.5–1.2%) with a modest degree of heterogeneity (PI 0.3–1.8%). Incidence of sNEC in premature infants (2.7%) and in studies from early surgical eras or with high risk of bias (2.1%) were significantly higher (*p* < 0.01). However, estimates of both groups had severe limitations. More than 90% of included patients and sNEC cases among premature infants were from a single study [46], which dominated the contribution of the only moderate risk of bias study in the group [26]. Incidence of sNEC in studies from early surgical eras or with high risk of bias ranged widely from a low of 0.6% to a high of 7.0%, secondary to heterogeneity in surgical era and method of ascertainment of sNEC cases, highlighting the unreliability of estimated sNEC incidence in this group.

For studies that reported it, surgical NEC as a proportion of all NEC ranged widely from 2 to 89%, and all estimates were adjudicated as high risk of bias. Using instead the pooled estimates of surgical NEC and all NEC from studies of moderate or low risk of bias, we estimated that the proportion of cardiogenic NEC which progresses to surgical NEC is 11% (0.8%/7.1%) in term infants and 21% (2.7%/13.0%) in premature infants.

### Risk Factors

**Fig. 5:**
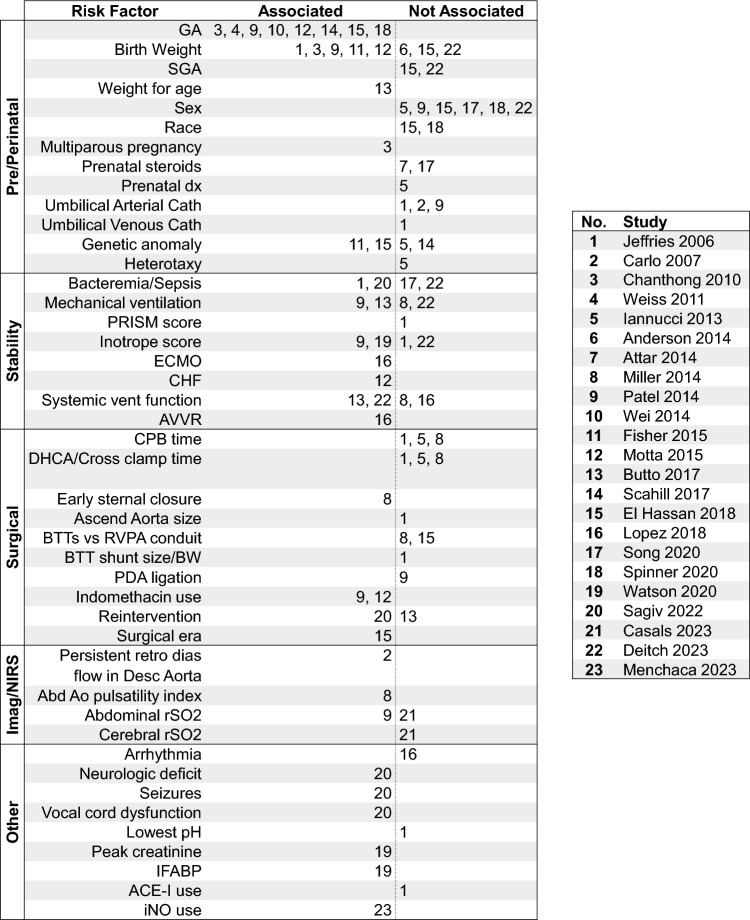
Studies (delineated by number in legend) of risk factors which found a positive association (*p* < 0.05) or no association with cardiogenic NEC in multivariable analysis

Twenty-three analyses investigating risk factors for cNEC evaluated a total of 43 unique risk factors (Fig. 5) [4, 27, 37, 43, 47, 48, 51, 52, 54, 56, 62, 63, 74, 76, 82, 87, 88, 90, 93, 94, 99, 100]. Only two risk factors clearly emerged as being consistently positively associated with cNEC: lower gestational age and lower birth weight. In eight of eight and five of eight studies which included gestational age and birth weight as covariates, a significant association was found with cNEC. Although birth weight is collinear with gestational age, it was an independent predictor of cNEC in three of four studies which included both covariates. In contrast, risk factors related to patient stability (e.g., inotrope score), surgical variables (e.g., cardiopulmonary bypass time), or imaging findings (e.g., abdominal aortic pulsatility index) were either investigated by too few studies or failed to demonstrate a consistent association with cNEC.

### Length of Stay

Eleven studies reported on hospital length of stay (LOS), with a slight majority (6) reporting median as opposed to mean LOS (Supplemental Table 5) [25, 27, 36, 42, 47, 63, 64, 72, 83, 93, 101]. Among the six studies which reported it, the median hospital LOS among CHD patients with NEC was 30.5 days (95% CI 10.5–59.5) longer than those without NEC. Of these studies, only one was moderate risk of bias [101], while the remainder were adjudicated as high risk of bias.

## Discussion

This systematic review and meta-analysis answers a number of fundamental questions about the burden of cardiogenic NEC in infants with structural congenital heart disease and reveals limitations in the extant literature to answer others. The estimated incidence of cardiogenic NEC among children with CHD was 7.1% in all term infants and 13.0% in low birth weight premature infants.

Given significant heterogeneity in study design, cNEC ascertainment, inclusion/exclusion criteria, and era effects, we chose to report incidences of cNEC in specific cardiac lesions as separate populations instead of as risk factors within the population of all CHD combined. Evidence for the incidence of cNEC in specific populations of cardiac anomalies was extremely limited. While the largest number of studies reported on patients with single ventricle physiology, only two were adjudicated as moderate risk of bias and none were low. A single, small study reported a 4.7% incidence of cNEC in all single ventricles, and a single, small study reported a 9.6% incidence in hybrid stage I patients. Such limited data preclude comparisons of cNEC incidence in these populations with all other forms of CHD.

We hypothesize that cNEC incidence differs between different cardiac lesions, but there is insufficient evidence to confirm this suspicion. All studies which reported incidences of cNEC in subgroups of anatomical abnormalities other than single ventricles were nearly universally adjudicated as high risk of bias. Estimates of cNEC incidence in these subgroups varied widely and should be interpreted within the context of their limitations. Similarly, there is inadequate evidence to conclude that diastolic runoff lesions in the form of ductal dependent physiology or Blalock–Taussig–Thomas shunts increase the risk of developing cNEC. Given the low level of certainty for even the pooled estimates of the available data, we do not recommend comparing the incidences of cNEC for specific cardiac lesions to identify risk factors. Instead, additional work is needed.

Evaluations of non-anatomic risk factors were numerous, but all studies investigating them were considered high risk of bias. Within those limitations, gestational age and birth weight were the only risk factors which consistently increased the risk of cNEC. We chose to exclude the role of feeding protocols on the risk of cNEC as multiple systematic reviews have addressed this topic [5–8]. Preoperative feeding was not associated with the development of cNEC in two meta-analyses, and there is some evidence to suggest that a primarily human breastmilk diet may be protective against cNEC.

We estimated that 11% of term and 21% of preterm infants with cardiogenic NEC ultimately progress to surgical intervention. Both estimates are substantially lower than what is reported in VLBW (52.1%) and ELBW (62.5%) infants of prematurity, corroborating the emerging consensus that the two are distinct clinical entities with different prognoses [102, 103]. However, even these estimates may seem high to current practitioners. The modern era’s increased monitoring, more sensitive imaging, and protocolized pre- and post-surgical management may lead to the increased detection of milder cases of cardiogenic NEC, increasing the number of NEC diagnoses but decreasing the proportion which require surgical intervention. In either event, that a much smaller proportion of cardiogenic NEC cases require surgery than in classical NEC of prematurity suggests that using the same diagnostic criteria (modified Bell’s) to initiate the same treatment strategy (broad spectrum antibiotics, NPO, and total parenteral nutrition) may be an overly conservative one-size fits all approach. Additional research is needed to determine whether risk factors can differentiate between infants with CHD who will and will not progress to clinical instability or a surgical intervention. This understanding would enable clinicians to tailor monitoring and treatment strategies according to specific risk profiles.

### Limitations

This systematic review and meta-analysis is most limited by the characteristics of the underlying studies and the small number of studies adjudicated as moderate or low risk of bias. Heterogeneity was minimal to non-existent among moderate or low risk of bias studies but universally high among high risk of bias studies. To mitigate the risk of pooling either unreliable estimates or estimates from studies with significantly different methodologies or populations, conclusions were drawn from pooled estimates of only moderate and low risk of bias studies where possible.

Studies reporting incidence and risk factors were the most beleaguered by unreliability in the ascertainment of NEC. In the modern era, the use of modified Bell’s criteria to diagnose and stage cNEC predominated. A minority of studies defined NEC operationally (e.g., use of NPO or antibiotics), which subjected the diagnosis of NEC to institutional practice variability in management. Repurposing modified Bell’s components from their original use as staging criteria to diagnostic criteria also has its limitations, as previously reported [104]. Some criteria may have both lower diagnostic importance to NEC and lower interrater reliability compared to others (e.g., absent bowel sounds vs portal venous gas). Further, modified Bell’s criteria do not specify which modality identifies the radiologic features despite evidence that ultrasound identifies pneumatosis and portal venous gas more frequently than x-ray [105]. Almost all studies were retrospective chart reviews, which is less reliable for certain criteria such as physical exam findings. A small minority of studies reported how radiographic images were obtained and verified, but no study reported a protocol for how clinical findings were abstracted or applied. Thus, there is a potential for heterogeneity not only between studies but within studies.

Studies reporting cNEC incidence generally fell into one of two categories: small, single-center chart reviews and large multicenter administrative database analyses. While small studies could conceivably review all notes and abdominal imaging, cohorts of thousands of infants were limited to queries of International Classification of Disease (ICD) billing codes whether this was explicitly stated or not. In this meta-analysis, studies which reported using billing codes to identify cNEC cases generally had lower—and in some cases substantially lower—incidences of cNEC, suggesting that billing codes are an insensitive method of ascertainment and likely lead to underestimation of cNEC burden. Indeed, the larger the cohort size the lower the reported incidence of NEC. Small studies, however, were limited by precision of the sample size. Assuming incidences of 7.1% and 0.5%, to achieve a precision of ± 25–50% a study would need to follow 200–800 and 1900–7600 participants to adequately estimate the incidences of cNEC and surgical NEC, respectively. Analyses of risk factors for NEC were severely limited by inadequate adjustment for all relevant confounders and by being underpowered.

The ascertainment of surgical NEC was considered more reliable than for medical NEC as operative records and procedure codes are more straightforward to extract and verify and require little to no interpretation. Studies of sNEC were most limited by small case numbers. One strength of meta-analysis is that the pooled estimate of multiple, relatively reliable studies yields a large enough case count to mitigate this limitation.

### Recommendations

The studies reviewed in this analysis highlight the challenges of estimating the burden of medical and surgical NEC in patients with CHD and suggest both best practices and directions for future research on cardiogenic NEC to consider.

Studies which report medical or surgical NEC as an outcome in CHD patients should specify the exact working definition (modified Bell’s or otherwise) but also provide a breakdown of which patients fulfill which criteria to optimize cross-compatibility with alternative staging systems and backwards compatibility with future staging systems. Definitions should focus on findings and not management decisions to minimize variability. Which imaging modality identified which radiographic features should be specified. Investigators should explicitly specify how patients were identified for chart review and further delineate how the chart was reviewed for cNEC criteria (e.g., regular expressions on radiology reports). Lastly, when reporting risk factors, non-randomized prophylactic interventions (e.g., probiotics), and outcomes (e.g., mortality), gestational age, birth weight, and anatomic subtype should be controlled for. Depending on the risk factor or population being investigated, additional confounders, including but not limited to those identified in this review, should be considered.

Future research is needed to identify which risk factors—either anatomic (e.g., single ventricles), physiologic (e.g., diastolic runoff), or innovative (e.g., blood tests or near infrared imaging)—can reliably predict which infants with CHD are most likely to develop long-term complications from NEC if no changes in management are undertaken. It is unclear whether all infants with CHD with pneumatosis on abdominal x-ray or ultrasound would progress to sepsis or laparotomy in the absence of stopping feeds and starting antibiotics. As infants with CHD already face a disproportionate risk of failure to thrive, the ability to identify which patients could safely continue enteral feeds holds significant clinical value.

## Supplementary Information

Below is the link to the electronic supplementary material.

Supplementary file1 (DOCX 1960 KB)

## Data Availability

No datasets were generated or analysed during the current study.

## Abbreviations

NEC: Necrotizing enterocolitis
cNEC: Cardiogenic necrotizing enterocolitis
sNEC: Surgical necrotizing enterocolitis
CHD: Congenital heart disease
CI: Confidence interval
PI: Prediction interval
PRISMA: Preferred reporting items for systematic reviews and meta-analyses
PDA: Patent ductus arteriosus
PFO: Patent foramen ovale
MeSH: Medical subject headings
RoB: Risk of bias
SWiM: Synthesis without meta-analysis
VLBW: Very low birth weight
ELBW: Extremely low birth weight
LOS: Length of stay
