# Supplementary material for: Cardiogenic Necrotizing Enterocolitis in Infants with Congenital Heart Disease: A Systematic Review and Meta-analysis
Source: Pediatr Cardiol. 2024 Oct 29;46(8):2429–42. doi: 10.1007/s00246-024-03686-4 (PMC12583363; doi:10.1007/s00246-024-03686-4)
Supplement: Supplementary file 1 — Supplementary file1 (DOCX 1960 KB) [file 246_2024_3686_MOESM1_ESM.docx]

**Supplemental Material**

Cardiogenic Necrotizing Enterocolitis in Infants with Congenital Heart Disease: A Systematic Review and Meta-Analysis

**Supplemental Table 1:**

|  | | | | | Contributed to Analysis | | | | | | |  |
| --- | --- | --- | --- | --- | --- | --- | --- | --- | --- | --- | --- | --- |
| Study | Country | Center no. | Study Design | Study Population | NEC Term/All | NEC Premature | NEC Single Ventricle | NEC Other Subgroup | Surgical NEC | Risk Factor | Length of Stay | Cohort Size |
| Dickinson 1982 | UK | Single center | Retrospective cohort study | Infants who required cardiac catheterization in the 1st month of life | + |  |  |  | + |  |  | 111 |
| Leung 1988 | Hong Kong | Single center | Retrospective cohort study | Symptomatic infants w/ CHD | + |  | + | + |  |  |  | 133 |
| Hebra 1993 | USA | Single center | Retrospective cohort study | Surgical Stage I (BTTS only) |  |  | + |  | + |  |  | 387 |
| Singh 1994 | UK | single center | Retrospective cohort study | Ductal dependent CHD |  |  |  | + |  |  |  | 34 |
| Sweet 1998 | UK | Single center | Retrospective cohort study | d-TGA+IVS or PA+IVS |  |  |  |  | + |  |  | 65 |
| Cheng 1999 | Hong Kong | Single center | Retrospective cohort study | Any CHD |  |  |  |  | + |  |  | 850 |
| Dees 2000 | USA | Single center | Retrospective cohort study | BW <1500 g, any CHD |  | + |  | + | + |  |  | 72 |
| McElhinney 2000 | USA | Single center | Retrospective cohort study | Admitted to cardiac ICU | + |  | + | + | + |  | + | 643 |
| Gillespie 2006 | USA | Single center | Retrospective cohort study | Required cardiac surgery, ≤6 months of age | + |  |  |  |  |  |  | 221 |
| Jeffries 2006 | USA | Single center | Retrospective cohort study | Surgical Stage I |  |  | + |  |  | + | + | 117 |
| Kelleher 2006 | USA | Single center | Retrospective cohort study | Surgical Stage I |  |  | + |  |  |  |  | 50 |
| Carlo 2007 | USA | Single center | Case-control study | Any CHD |  |  |  |  |  | + |  | 38 |
| Lambert 2007 | USA | Multicenter | Retrospective cohort study | CHD patients admitted to NICU, GA ≥36 weeks | + |  |  |  |  |  |  | 278 |
| Tweddel 2007 | USA | Single center | Retrospective cohort study | Surgical Stage I |  |  | + |  |  |  |  | 178 |
| Willis 2008 | USA | Single center | Retrospective cohort study | Ductal dependent CHD |  |  |  | + |  |  |  | 33 |
| Braudis 2009 | USA | Single center | Prospective cohort study | Surgical Stage I |  |  | + |  |  |  |  | 78 |
| Ades 2010 | USA | Single center | Retrospective cohort study | BW <2500g, required cardiac surgery |  | + |  |  |  |  |  | 105 |
| Chanthong 2010 | Canada | Single center | Retrospective cohort study | Admitted to cardiac ICU | + |  |  |  |  | + | + | 1185 |
| Del Castillo 2010 | USA | Single center | Retrospective cohort study | Surgical Stage I |  |  | + |  |  |  | + | 98 |
| Mukherjee 2010 | USA | Multicenter | Retrospective cohort study | Required cardiac surgery | + |  | + | + |  |  |  | 11958 |
| Luce 2011 | USA | Single center | Retrospective cohort study | Hybrid Stage I |  |  |  |  |  |  | + | 73 |
| Natarajan 2011 | USA | Single center | Retrospective cohort study | GA ≥ 34 weeks, required cardiac surgery | + | + |  |  |  |  |  | 88 |
| Weiss 2011 | USA | Single center | Retrospective cohort study | Single ventricle |  |  | + |  |  | + |  | 32 |
| Pappas 2012 | USA | Multicenter | Retrospective cohort study | BW 400-1000g, any CHD and no other congenital issues |  | + |  |  |  |  |  | 105 |
| Davies 2013 | USA | Single center | Retrospective cohort study | Single ventricle |  |  | + |  |  |  | + | 15 |
| Dilli 2013 | Turkey | Single center | Randomized controlled trial | Cyanotic CHD, GA ≥35 weeks | + |  |  |  | + |  |  | 100 |
| Iannucci 2013 | USA | Single center | Retrospective cohort study | Any CHD | + |  |  |  |  | + |  | 1551 |
| Kargl 2013 | Austria | Single center | Retrospective cohort study | Required cardiac surgery | + |  |  |  | + |  |  | 754 |
| Anderson 2014 | USA | Single center | Retrospective cohort study | BW <1500 g, GA <32 weeks, major CHD |  | + |  |  |  | + |  | 299 |
| Attar 2014 | USA | Single center | Retrospective cohort study | GA 34 0/7 - 36 6/7 weeks, complex CHD |  | + |  |  |  | + |  | 106 |
| Cozzi 2014 | USA | Single center | Prospective cohort study | Hybrid Stage I |  |  | + |  |  |  |  | 15 |
| Bain 2014 | USA | Single center | Retrospective cohort study | GA <37 weeks |  | + |  | + |  |  |  | 3993 |
| Dewitt 2014 | USA | Single center | Prospective cohort study | Single ventricle |  |  | + | + |  |  |  | 34 |
| Kalfa 2014 | USA | Single center | Retrospective cohort study | BW <2.5 kg, required cardiac surgery, ≤12 months old |  | + |  |  |  |  |  | 146 |
| Miller 2014 | USA | Single center | Retrospective cohort study | Surgical Stage I |  |  | + |  |  | + | + | 61 |
| Patel 2014 | USA | Single center | Prospective cohort study | GA <32 weeks, BW <1500 g, any CHD, ≤12 months old |  |  |  |  |  |  | + | 100 |
| Wei 2014 | USA | Single center | Retrospective cohort study | BW <2.5 kg, any CHD, ≤12 months old |  | + |  |  |  | + |  | 74 |
| Alten 2015 | USA | Multicenter | Retrospective cohort study | Required cardiac surgery | + |  |  |  |  |  |  | 251 |
| Motta 2015 | USA | Single center | Retrospective cohort study | GA 23/0 to 34/6, any CHD |  | + |  | + |  | + |  | 170 |
| Fisher 2015 | USA | Multicenter | Retrospective cohort study | BW <1.5 kg with severe CHD |  | + |  | + |  | + |  | 1931 |
| Carpenito 2016 | USA | Single center | Retrospective cohort study | Hybrid Stage I |  |  | + |  |  |  |  | 125 |
| Iliopoulos 2016 | UK | Single center | Prospective cohort study | <10 kg at time of cardiac surgery | + |  |  |  |  |  |  | 50 |
| Butto 2017 | USA | Single center | Retrospective cohort study | Surgical Stage I (BTTS only) |  |  | + |  | + | + |  | 18 |
| Cua 2017 | USA | Multicenter | Retrospective cohort study | T21, any CHD |  |  |  | + |  |  |  | 3957 |
| Ferguson 2017 | Australia | Single center | Retrospective cohort study | GA <32 weeks, ductal dependent CHD |  |  |  | + | + |  |  | 14 |
| Manuri 2017 | Italy | Single center | Retrospective cohort study | Hybrid Stage I |  |  | + |  |  |  |  | 42 |
| Scahill 2017 | USA | Single center | Retrospective cohort study | Required cardiac surgery | + |  |  |  | + | + |  | 130 |
| Tadphale 2017 | USA | Multicenter | Retrospective cohort study | Surgical Stage I |  |  | + |  |  |  |  | 5374 |
| Avram 2018 | USA | Single center | Retrospective cohort study | 22q11, any CHD |  |  |  | + |  |  |  | 35 |
| ElHassan 2018 | USA | Multicenter | Retrospective cohort study | Single ventricle |  |  | + |  | + | + |  | 5720 |
| Fuchs 2018 | USA | Single center | Retrospective cohort study | Turner, HLHS |  |  |  | + |  |  |  | 6 |
| Lau 2018 | USA | Single center | Retrospective cohort study | Any CHD | + |  | + | + | + |  |  | 1811 |
| Lopez 2018 | USA | Multicenter | Retrospective cohort study | Surgical Stage I |  |  | + |  |  | + | + | 1163 |
| Nellihela 2018 | UK | Single center | Retrospective cohort study | Required cardiac surgery or transcutaneous intervention | + |  |  |  |  |  |  | 1608 |
| Schuchardt 2018 | USA | Single center | Retrospective cohort study | Required cardiac surgery, ≤6 months old | + |  |  |  |  |  | + | 251 |
| Day 2019 | Australia | Single center | Retrospective cohort study | Ductal dependent CHD |  |  |  | + |  |  |  | 177 |
| Santro 2019 | Australia | Single center | Retrospective cohort study | Single ventricle |  |  | + |  |  |  |  | 2019 |
| Stockley 2019 | UK | Single center | Retrospective cohort study | Truncus/Aortopulmonary window |  |  |  | + |  |  |  | 15 |
| Hamzah 2020 | USA | Multicenter | Retrospective cohort study | Truncus/Aortopulmonary window |  |  |  | + |  |  |  | 3010 |
| Kocjancic 2020 | Germany | Single center | Retrospective cohort study | Ductal dependent CHD |  |  | + | + | + |  |  | 488 |
| Norman 2020 | Intl | Multicenter | Retrospective cohort study | GA 24-31 weeks, BW <1500g, major CHD |  | + |  |  |  |  |  | 609 |
| Nordenstrom 2020 | Sweden | Single center | Retrospective cohort study | Infants requiring surgery or transcutaneous intervention within 2 months of life | + |  |  |  | + |  |  | 458 |
| O'Neal 2020 | USA | Single center | Retrospective cohort study | Infants who required cardiac surgery | + |  |  |  |  |  |  | 570 |
| Raymond 2020 | USA | Single center | Retrospective cohort study | Single ventricle |  |  | + |  |  |  |  | 63 |
| Song 2020 | South Korea | Single center | Retrospective cohort study | BW ≤1500 g, any CHD |  | + |  |  | + | + |  | 72 |
| Spinner 2020 | USA | Multicenter | Retrospective cohort study | Any CHD | + |  | + | + |  | + | + | 3877 |
| van der Heide 2020 | Nether. | Single center | Retrospective cohort study | GA ≥35 weeks, any CHD, ≤12 months of age | + |  |  |  |  |  |  | 956 |
| Watson 2020 | USA | Single center | Retrospective cohort study | Required cardiac surgery with 120 days of life, BW >2 kg, GA >34 weeks | + |  |  |  |  | + |  | 108 |
| Cheung 2021 | Canada | Single center | Retrospective cohort study | GA <37 weeks, complex CHD |  | + |  |  |  |  |  | 115 |
| Kataria-Hale 2021 | USA | Single center | Retrospective cohort study | Ductal dependent CHD |  |  |  | + |  |  |  | 408 |
| Kirli 2021 | Turkey | Single center | Retrospective cohort study | Required cardiac surgery |  |  |  |  | + |  |  | 164 |
| Moza 2021 | USA | Multicenter | Retrospective cohort study | s/p Stage I, ≤12 months of age |  |  |  |  | + |  |  | 730 |
| Boos 2022 | Germany | Single center | Retrospective cohort study | TGA |  |  |  | + |  |  |  | 100 |
| Choi 2022 | South Korea | Single center | Retrospective cohort study | GA ≥37 weeks, ductal dependent CHD, ≤12 months of age |  |  |  | + | + |  |  | 355 |
| Dumitrascu 2022 | UK | Single center | Retrospective cohort study | GA ≤37 weeks, any CHD |  | + |  |  |  |  |  | 115 |
| Hernstadt 2022 | UK | Single center | Retrospective cohort study | Complete heart block |  |  |  | + |  |  |  | 29 |
| Menchaca 2022 | USA | Single center | Retrospective cohort study | Complex CHD |  |  |  | + | + |  |  | 322 |
| Pham 2022 | USA | Single center | Retrospective cohort study | Required cardiac surgery |  |  |  |  |  |  | + | 282 |
| Sagiv 2022 | USA | Multicenter | Retrospective cohort study | Single ventricle |  |  | + |  |  | + |  | 1314 |
| Tan 2022 | China | Single center | Retrospective cohort study | BW 1500-2500g, any CHD |  | + |  |  |  |  |  | 18 |
| Blanco 2023 | USA | Single center | Randomized controlled trial | Single ventricle |  |  | + |  |  |  |  | 107 |
| Casals 2023 | USA | Single center | Retrospective cohort study | Required cardiac surgery | + |  |  |  |  | + |  | 166 |
| Deitch 2023 | USA | Single center | Retrospective cohort study | GA ≥ 37 weeks, admitted to cardiac ICU | + |  |  |  | + | + |  | 3933 |
| Fujita 2023 | USA | Single center | Retrospective cohort study | Post-op pulmonary vein stenosis |  |  |  | + |  |  |  | 29 |
| Menchaca 2023 | USA | Single center | Retrospective cohort study | Complex CHD | + |  |  |  |  | + |  | 352 |
| Penk 2023 | USA | Single center | Retrospective cohort study | Ductal dependent CHD |  |  |  | + |  |  |  | 127 |

Study characteristics of all included studies. NEC, necrotizing enterocolitis; CHD, congenital heart disease; BTTS, Blalock-Taussig-Thomas shunt; TGA, transposition of the great arteries; PA, pulmonary atresia; IVS, intact ventricular septum; GA, gestational age; BW, birth weight

**Supplemental Table 2:**

| Study | Risk of bias | Sample frame appropriate to address the target population? | Study participants sampled appropriately? | Sample size adequate? | Study subjects and setting described in detail? | Coverage bias? | Valid methods used for identification of condition? | Condition measured in a standard, reliable way for all participants? | Statistical analysis appropriate? | Response rate adequate or low response rate managed appropriately? |
| --- | --- | --- | --- | --- | --- | --- | --- | --- | --- | --- |
| Dickinson 1982 | High | - | + | - | + | n/a | - | - | + | n/a |
| Leung 1988 | Mod | + | + | +/- | + | n/a | +/- | + | + | n/a |
| McElhinney 2000 | High | + | + | +/- | + | n/a | - | + | + | n/a |
| Gillespie 2006 | High | + | + | +/- | + | n/a | - | - | + | n/a |
| Lambert 2007 | High | + | + | +/- | + | n/a | - | + | + | n/a |
| Chanthong 2010 | High | + | +/- | + | - | n/a | - | - | + | n/a |
| Mukherjee 2010 | High | + | +/- | + | + | n/a | - | - | + | n/a |
| Natarajan 2011 | High | + | +/- | - | +/- | n/a | - | - | + | n/a |
| Dilli 2013 | Mod | + | + | - | + | n/a | +/- | +/ | + | n/a |
| Iannucci 2013 | High | + | +/- | + | +/- | n/a | - | - | + | n/a |
| Kargl 2013 | High | + | - | +/- | - | n/a | - | - | + | n/a |
| Alten 2015 | High | - | + | +/- | + | n/a | - | + | + | n/a |
| Iliopoulos 2016 | High | +/- | - | - | + | n/a | - | - | + | n/a |
| Scahill 2017 | High | + | + | - | + | n/a | + | - | + | n/a |
| Lau 2018 | High | + | + | + | + | n/a | - | - | + | n/a |
| Nellihela 2018 | High | +/- | - | + | - | n/a | - | - | + | n/a |
| Schuchardt 2018 | Mod | - | - | + | + | n/a | + | + | + | n/a |
| Nordenstrom 2020 | High | + | + | + | + | n/a | - | - | + | n/a |
| O'Neal 2020 | High | + | + | + | + | n/a | - | - | + | n/a |
| Spinner 2020 | High | + | + | + | + | n/a | - | - | + | n/a |
| van der Heide 2020 | High | + | + | + | + | n/a | - | - | - | n/a |
| Watson 2020 | High | +/- | + | - | + | n/a | - | - | + | n/a |
| Casals 2023 | High | + | + | - | - | n/a | - | - | - | n/a |
| Deitch 2023 | High | + | + | + | - | n/a | - | - | + | n/a |
| Menchaca 2023 | Mod | + | +/- | +/- | - | n/a | - | - | + | n/a |

Risk of bias table for studies reporting incidence of cardiogenic NEC in cohorts of all infants with all forms of congenital heart disease combined. Described study methodologies either adequately (+), partially (+/-), or inadequately (-) address the potential sources of bias. The overall assessment of risk of bias is not composite score of the underlying criteria but a holistic evaluation.

**Supplemental Table 3:**

| Study | Risk of bias | Sample frame appropriate to address the target population? | Study participants sampled appropriately? | Sample size adequate? | Study subjects and setting described in detail? | Coverage bias? | Valid methods used for identification of condition? | Condition measured in a standard, reliable way for all participants? | Statistical analysis appropriate? |
| --- | --- | --- | --- | --- | --- | --- | --- | --- | --- |
| Dees 2000 | High | + | + | +/- | + | n/a | - | - | + |
| Ades 2010 | High | + | + | - | + | n/a | - | - | + |
| Natarajan 2011 | High | + | +/- | - | +/- | n/a | - | - | + |
| Pappas 2012 | Low | + | + | +/- | + | n/a | + | + | + |
| Anderson 2014 | High | + | + | + | +/- | n/a | - | - | + |
| Attar 2014 | High | - | + | - | + | n/a | - | - | + |
| Kalfa 2014 | High | + | + | - | + | n/a | - | - | + |
| Wei 2014 | High | + | + | - | + | n/a | - | - | + |
| Motta 2015 | Mod | + | + | - | + | n/a | + | + | + |
| Fisher 2015 | Low | + | + | + | + | n/a | + | + | + |
| Norman 2020 | High | + | + | + | + | n/a | - | - | + |
| Song 2020 | Mod | + | + | - | + | n/a | +/- | + | + |
| Cheung 2021 | High | + | + | - | + | n/a | - | - | + |
| Dumitrascu 2022 | High | + | + | + | - | n/a | - | - | + |
| Tan 2022 | High | + | + | - | +/- | n/a | - | - | + |

Risk of Bias Table for studies reporting incidence of cardiogenic NEC in cohorts of exclusively premature infants with all forms of congenital heart disease combined. Described study methodologies either adequately (+), partially (+/-), or inadequately (-) address the potential sources of bias. The overall assessment of risk of bias is not composite score of the underlying criteria but a holistic evaluation.

| Study | Risk of bias | Sample frame appropriate to address the target population? | Study participants sampled appropriately? | Sample size adequate? | Study subjects and setting described in detail? | Coverage bias? | Valid methods used for identification of condition? | Condition measured in a standard, reliable way for all participants? | Statistical analysis appropriate? | Response rate adequate or low response rate managed appropriately? |
| --- | --- | --- | --- | --- | --- | --- | --- | --- | --- | --- |
| Dickinson 1982 | Mod | - | + | - | + | n/a | + | + | + | n/a |
| Hebra 1993 | Low | - | + | +/- | + | n/a | +/- | + | + | n/a |
| Sweet 1998 | Mod | - | + | - | + | n/a | + | + | + | n/a |
| Cheng 1999 | Low | - | + | + | + | n/a | +/- | + | + | n/a |
| Dees 2000 | Mod | + | + | - | + | n/a | + | + | + | n/a |
| McElhinney 2000 | Mod | + | + | - | + | n/a | + | + | + | n/a |
| Dilli 2013 | High | +/- | + | - | + | n/a | - | - | + | n/a |
| Kargl 2013 | Low | + | - | +/- | - | n/a | +/- | +/- | + | n/a |
| Bain 2014 | High | +/- | + | + | - | n/a | +/- | - | + | n/a |
| Butto 2017 | Mod | +/- | + | - | - | n/a | + | + | + | n/a |
| Ferguson 2017 | High | +/- | - | - | - | n/a | - | - | + | n/a |
| Scahill 2017 | Mod | + | + | - | + | n/a | + | + | + | n/a |
| ElHassan 2018 | Low | +/- | + | + | + | n/a | +/- | +/- | + | n/a |
| Lau 2018 | Low | + | + | + | + | n/a | + | + | + | n/a |
| Kocjancic 2020 | Low | +/- | - | - | + | n/a | +/- | +/- | + | n/a |
| Nordenstrom 2020 | Mod | + | + | - | + | n/a | + | + | + | n/a |
| Song 2020 | High | + | + | - | + | n/a | - | - | + | n/a |
| Kirli 2021 | Mod | + | + | - | - | n/a | - | - | + | n/a |
| Moza 2021 | Mod | +/- | + | - | - | n/a | - | - | + | n/a |
| Choi 2022 | High | +/- | - | - | - | n/a | +/- | - | + | n/a |
| Menchaca 2022 | High | + | +/- | +/- | +/- | n/a | - | - | + | n/a |
| Deitch 2023 | Low | + | + | + | + | n/a | + | + | + | n/a |

**Supplemental Table 4:**

Risk of bias table for studies reporting incidence of surgical cardiogenic NEC. Described study methodologies either adequately (+), partially (+/-), or inadequately (-) address the potential sources of bias. The overall assessment of risk of bias is not composite score of the underlying criteria but a holistic evaluation.

**Supplemental Figure 1:**

**
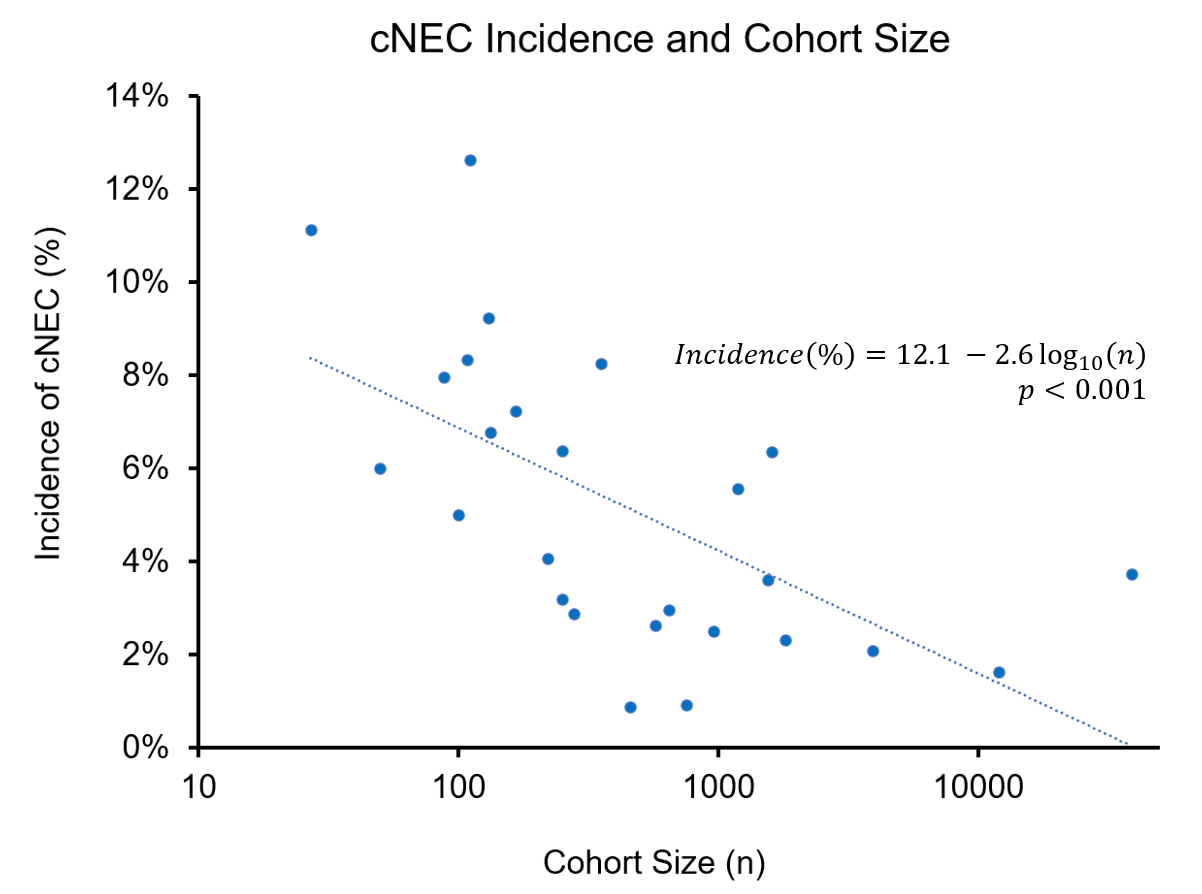
**

Relationship between cohort size and prevalence of NEC. NB: x-axis is on a base-10 logarithmic scale.

**Supplemental Figure 2**: Forest plot of Cardiogenic NEC incidence in Single Ventricle patients


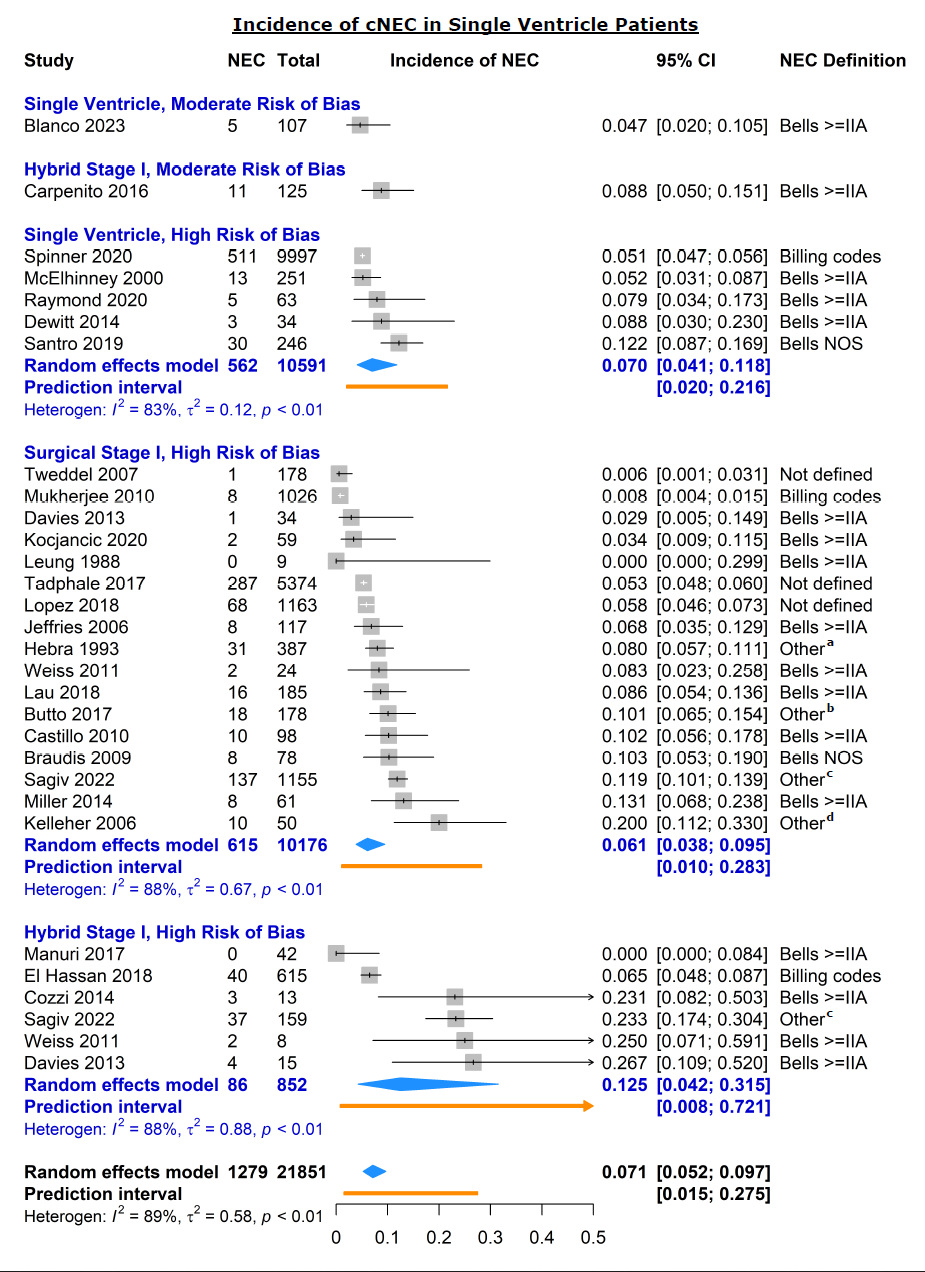


Individual (squares) and pooled (diamonds) estimates of incidence of cardiogenic NEC in infants with single ventricles. ^a^Two of three: clinical, radiographic, or pathologic signs of NEC; ^b^Clinical findings which required antibiotics or abdominal surgery; ^c^Differed by registry site; ^d^Clinical or radiographic findings requiring NPO

**Supplemental Figure 3:** Forest plot of Cardiogenic NEC incidence by cardiac lesion in term infants


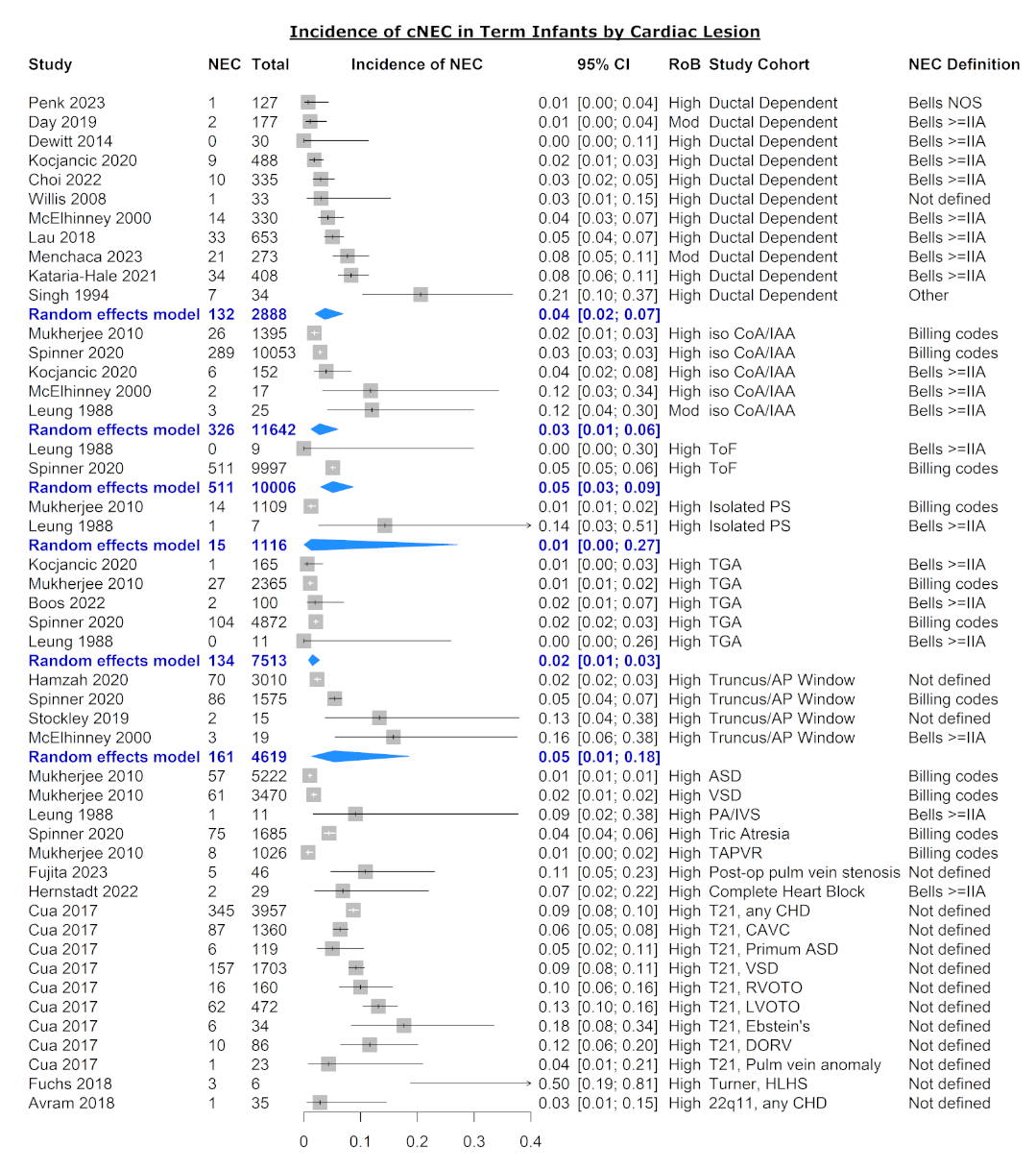


Individual (squares) and pooled (diamonds) estimates of incidence of cardiogenic NEC in all or term infants by cardiac anomaly excluding single ventricles. RoB, risk of bias; CoA, coarctation of aorta; IAA, interrupted aortic arch; ToF, Tetralogy of Fallot; PS, pulmonary stenosis; TGA, transposition of the great arteries; AP, aortopulmonary; ASD, atrial septal defect; VSD, ventricular septal defect; PA, pulmonary atresia; IVS, intact ventricular septum; TAPVR, total anomalous pulmonary venous return; CHD, congenital heart disease; CAVC, common atrioventricular canal; RVOTO, right ventricular outflow tract obstruction; LVOTO; left ventricular outflow tract obstruction; DORV, double outlet right ventricle; HLHS, hypoplastic left heart syndrome; (1) clinical and radiographic signs (including distended small bowel loops)

**Supplemental Figure 4:** Forest plot of cardiogenic NEC incidence in premature infants within a subgroup of congenital heart disease stratified by risk of bias


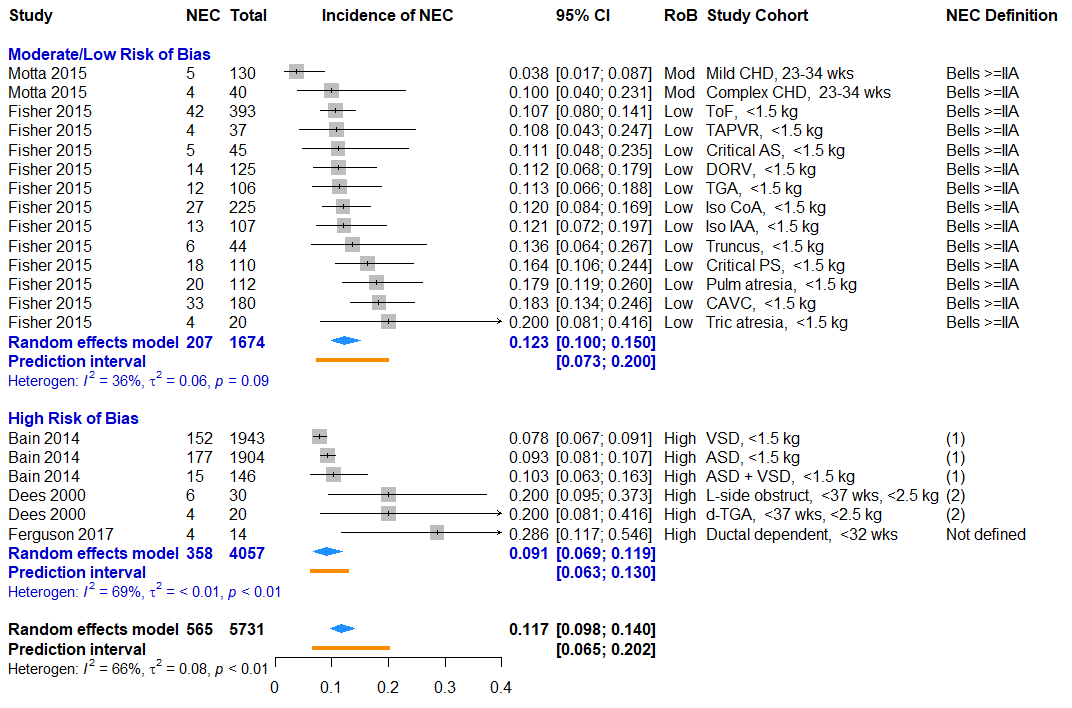


Individual (squares) and pooled (diamonds) estimates of incidence of cardiogenic NEC in premature infants by cardiac anomaly excluding single ventricles. RoB, risk of bias; CHD, congenital heart disease; wks, gestational age in weeks; kg, birth weight in kilograms. Complex CHD excludes isolated atrial and ventricular septal defects. (1), Internal administrative database; (2), each of: clinical signs, x-ray findings, and required antibiotics or NPO

**Supplemental Table 5:** Hospital Length of Stay by NEC in CHD patients stratified by outcome metric (median vs mean LOS)

|  | NEC | | No NEC | |  |
| --- | --- | --- | --- | --- | --- |
| Study | Median LOS (IQR)  days | n | Median LOS (IQR)  days | n | Difference  (days) |
| Del Castillo 2010 | 55 (34, 83) | 17 | 21 (16, 36) | 81 | 34 |
| Luce 2011 | 35 (8, 49) | 8 | 19 (9, 100) | 65 | 16 |
| Lopez 2018 | 55 (39, 74) | 57 | 28 (21, 42) | 171 | 27 |
| Schuchardt 2018 | 53 (36, 93) | 16 | 10 (6, 23) | 217 | 43 |
| Spinner 2020 | 54 (NR) | 1,448 | 18 (not reported) | 37,322 | 36 |
| Chanthong 2010 | 10 (NR) | 66 | 5 (not reported) | 1119 | 5 |
|  |  |  |  |  | 30.5 (10.5-39.5) |
|  |  |  |  |  |  |
|  | Mean LOS (SD)  days |  | Mean LOS (SD)  days |  | Difference  (days) |
| McElhinney 2000 | 36 (22) | 21 | 19 (14) | 622 | 17 |
| Jeffries 2006 | 59 (36) | 9 | 29 (32) | 96 | 30 |
| Davies 2013 | 89.5 (54.2) | 11 | 39.6 (26.7) | 49 | 49 |
| Miller 2014 | 31.6 (36.4) | 30 | 22.7 (7.6) | 50 | 8.9 |
| Pham 2022 | 66.6 (44.7) | 21 | 40.6 (42.9) | 50 | 26.0 |
|  |  |  |  |  | 21.1 (12.1, 30.0) |

NR, not reported.

**Supplemental Methods 1:** Search Logic for Medline and Embase databases

**Medline search strategy:**

Search: ((infant) AND ((((((((congenital heart disease) OR (congenital heart defect)) OR ((cardiology service, hospital[MeSH Terms]) OR (cardiology hospital services[MeSH Terms]))) OR (cardiovascular surgical procedure[MeSH Terms])) OR (cardiopulmonary bypass)) OR (extracorporeal circulation[MeSH Terms])) OR (hemodynamics[MeSH Terms])) OR ((((((treatment out[MeSH Terms]) OR (postoperative complication[MeSH Terms])) OR (intensive care units, pediatric[MeSH Terms])) OR (intensive care, neonatal[MeSH Terms]))) AND ((cardiovascular disease[MeSH Terms]) OR (heart[MeSH Terms]))))) AND ((((((necrotizing enterocolitis) OR (gastrointestinal hemorrhage)) OR (hematochezia)) OR (splanchnic circulation[MeSH Terms])) OR (intestinal mucosa[MeSH Terms])) OR (((spectroscopy, near infrared[MeSH Terms]) OR (occult blood[MeSH Terms])) AND (((somatic) OR (gastrointestinal tract[MeSH Terms])) OR (gastrointestinal disease[MeSH Terms])))) Sort by: Publication Date

(((("infant"[MeSH Terms] OR "infant"[All Fields]) OR "infants"[All Fields]) OR "infant s"[All Fields]) AND ((((((((("congenit heart dis"[Journal] OR (("congenital"[All Fields] AND "heart"[All Fields]) AND "disease"[All Fields])) OR "congenital heart disease"[All Fields]) OR (((("heart defects, congenital"[MeSH Terms] OR (("heart"[All Fields] AND "defects"[All Fields]) AND "congenital"[All Fields])) OR "congenital heart defects"[All Fields]) OR (("congenital"[All Fields] AND "heart"[All Fields]) AND "defect"[All Fields])) OR "congenital heart defect"[All Fields])) OR ("cardiology service, hospital"[MeSH Terms] OR "cardiology service, hospital"[MeSH Terms])) OR "cardiovascular surgical procedures"[MeSH Terms]) OR (("cardiopulmonary bypass"[MeSH Terms] OR ("cardiopulmonary"[All Fields] AND "bypass"[All Fields])) OR "cardiopulmonary bypass"[All Fields])) OR "extracorporeal circulation"[MeSH Terms]) OR "hemodynamics"[MeSH Terms]) OR ((("postoperative complications"[MeSH Terms] OR "intensive care units, pediatric"[MeSH Terms]) OR "intensive care, neonatal"[MeSH Terms]) AND ("cardiovascular diseases"[MeSH Terms] OR "heart"[MeSH Terms])))) AND ((((((((("necrotising enterocolitis"[All Fields] OR "enterocolitis, necrotizing"[MeSH Terms]) OR ("enterocolitis"[All Fields] AND "necrotizing"[All Fields])) OR "necrotizing enterocolitis"[All Fields]) OR ("necrotizing"[All Fields] AND "enterocolitis"[All Fields])) OR ((("gastrointestinal haemorrhage"[All Fields] OR "gastrointestinal hemorrhage"[MeSH Terms]) OR ("gastrointestinal"[All Fields] AND "hemorrhage"[All Fields])) OR "gastrointestinal hemorrhage"[All Fields])) OR (((("gastrointestinal hemorrhage"[MeSH Terms] OR ("gastrointestinal"[All Fields] AND "hemorrhage"[All Fields])) OR "gastrointestinal hemorrhage"[All Fields]) OR "haematochezia"[All Fields]) OR "hematochezia"[All Fields])) OR "splanchnic circulation"[MeSH Terms]) OR "intestinal mucosa"[MeSH Terms]) OR (("spectroscopy, near-infrared"[MeSH Terms] OR "occult blood"[MeSH Terms]) AND (((((("diploidy"[MeSH Terms] OR "diploidy"[All Fields]) OR "somatic"[All Fields]) OR "somatically"[All Fields]) OR "somatics"[All Fields]) OR "gastrointestinal tract"[MeSH Terms]) OR "gastrointestinal diseases"[MeSH Terms])))

Translations

infant: "infant"[MeSH Terms] OR "infant"[All Fields] OR "infants"[All Fields] OR "infant's"[All Fields]

congenital heart disease: "Congenit Heart Dis"[Journal:__jid101256510] OR ("congenital"[All Fields] AND "heart"[All Fields] AND "disease"[All Fields]) OR "congenital heart disease"[All Fields]

congenital heart defect: "heart defects, congenital"[MeSH Terms] OR ("heart"[All Fields] AND "defects"[All Fields] AND "congenital"[All Fields]) OR "congenital heart defects"[All Fields] OR ("congenital"[All Fields] AND "heart"[All Fields] AND "defect"[All Fields]) OR "congenital heart defect"[All Fields]

cardiology service, hospital[MeSH Terms]: "cardiology service, hospital"[MeSH Terms]

cardiology hospital services[MeSH Terms]: "cardiology service, hospital"[MeSH Terms]

cardiovascular surgical procedure[MeSH Terms]: "cardiovascular surgical procedures"[MeSH Terms]

cardiopulmonary bypass: "cardiopulmonary bypass"[MeSH Terms] OR ("cardiopulmonary"[All Fields] AND "bypass"[All Fields]) OR "cardiopulmonary bypass"[All Fields]

extracorporeal circulation[MeSH Terms]: "extracorporeal circulation"[MeSH Terms]

hemodynamics[MeSH Terms]: "hemodynamics"[MeSH Terms]

postoperative complication[MeSH Terms]: "postoperative complications"[MeSH Terms]

intensive care units, pediatric[MeSH Terms]: "intensive care units, pediatric"[MeSH Terms]

intensive care, neonatal[MeSH Terms]: "intensive care, neonatal"[MeSH Terms]

cardiovascular disease[MeSH Terms]: "cardiovascular diseases"[MeSH Terms]

heart[MeSH Terms]: "heart"[MeSH Terms]

necrotizing enterocolitis: "necrotising enterocolitis"[All Fields] OR "enterocolitis, necrotizing"[MeSH Terms] OR ("enterocolitis"[All Fields] AND "necrotizing"[All Fields]) OR "necrotizing enterocolitis"[All Fields] OR ("necrotizing"[All Fields] AND "enterocolitis"[All Fields])

gastrointestinal hemorrhage: "gastrointestinal haemorrhage"[All Fields] OR "gastrointestinal hemorrhage"[MeSH Terms] OR ("gastrointestinal"[All Fields] AND "hemorrhage"[All Fields]) OR "gastrointestinal hemorrhage"[All Fields]

hematochezia: "gastrointestinal hemorrhage"[MeSH Terms] OR ("gastrointestinal"[All Fields] AND "hemorrhage"[All Fields]) OR "gastrointestinal hemorrhage"[All Fields] OR "haematochezia"[All Fields] OR "hematochezia"[All Fields]

splanchnic circulation[MeSH Terms]: "splanchnic circulation"[MeSH Terms]

intestinal mucosa[MeSH Terms]: "intestinal mucosa"[MeSH Terms]

spectroscopy, near infrared[MeSH Terms]: "spectroscopy, near-infrared"[MeSH Terms]

occult blood[MeSH Terms]: "occult blood"[MeSH Terms]

somatic: "diploidy"[MeSH Terms] OR "diploidy"[All Fields] OR "somatic"[All Fields] OR "somatically"[All Fields] OR "somatics"[All Fields]

gastrointestinal tract[MeSH Terms]: "gastrointestinal tract"[MeSH Terms]

gastrointestinal disease[MeSH Terms]: "gastrointestinal diseases"[MeSH Terms]

**EMBASE search strategy:**

('infant'/exp OR 'infant' OR 'infants'/exp OR 'infants')

AND

((‘congenital heart disease’/exp OR (‘congenital’ AND ‘heart’ AND ‘disease’) OR ‘congenital heart disease’ OR ((‘heart’ AND ‘defects’) AND ‘congenital’) OR ‘congenital heart defect’ OR ((‘congenital’ AND ‘heart’) AND ‘defect’))

OR ‘cardiology service’/exp OR ‘cardiovascular surgery’/exp

OR ((‘cardiopulmonary bypass’/exp OR (‘cardiopulmonary’ AND ‘bypass’) OR ‘cardiopulmonary bypass’)))

AND

((‘necrotizing enterocolitis’ OR ‘necrotizing enterocolitis’/exp OR (‘enterocolitis’ AND ‘necrotizing’) OR ‘necrotizing enterocolitis’ OR (‘necrotizing’ AND ‘enterocolitis’))

OR (‘gastrointestinal haemorrhage’ OR ‘gastrointestinal hemorrhage’/exp OR (‘gastrointestinal’ AND ‘hemorrhage’))

OR (‘haematochezia’ OR ‘hematochezia’ OR ‘splanchnic blood flow’/exp))

**Supplemental Methods 2:** Search Logic for Medline and Embase databases


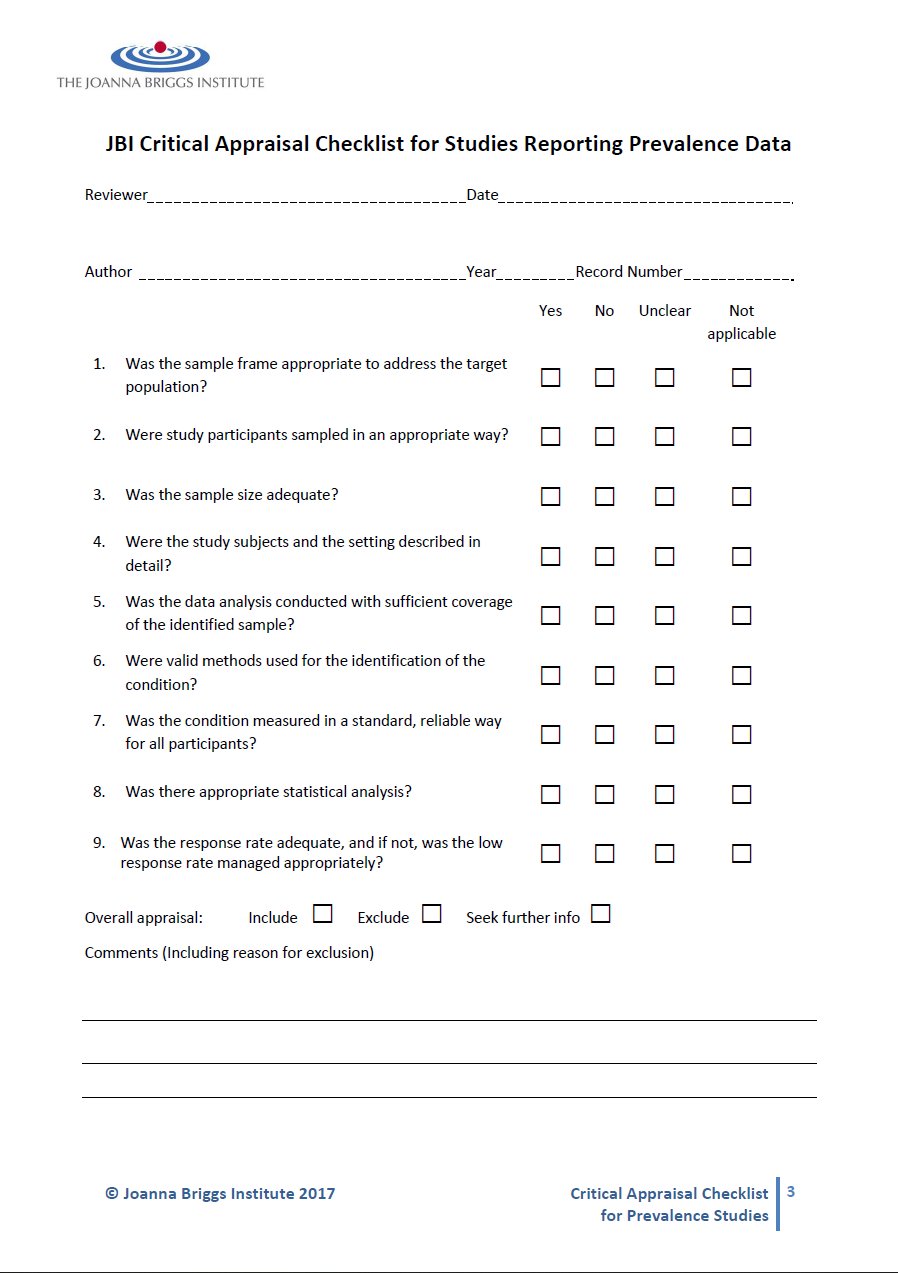


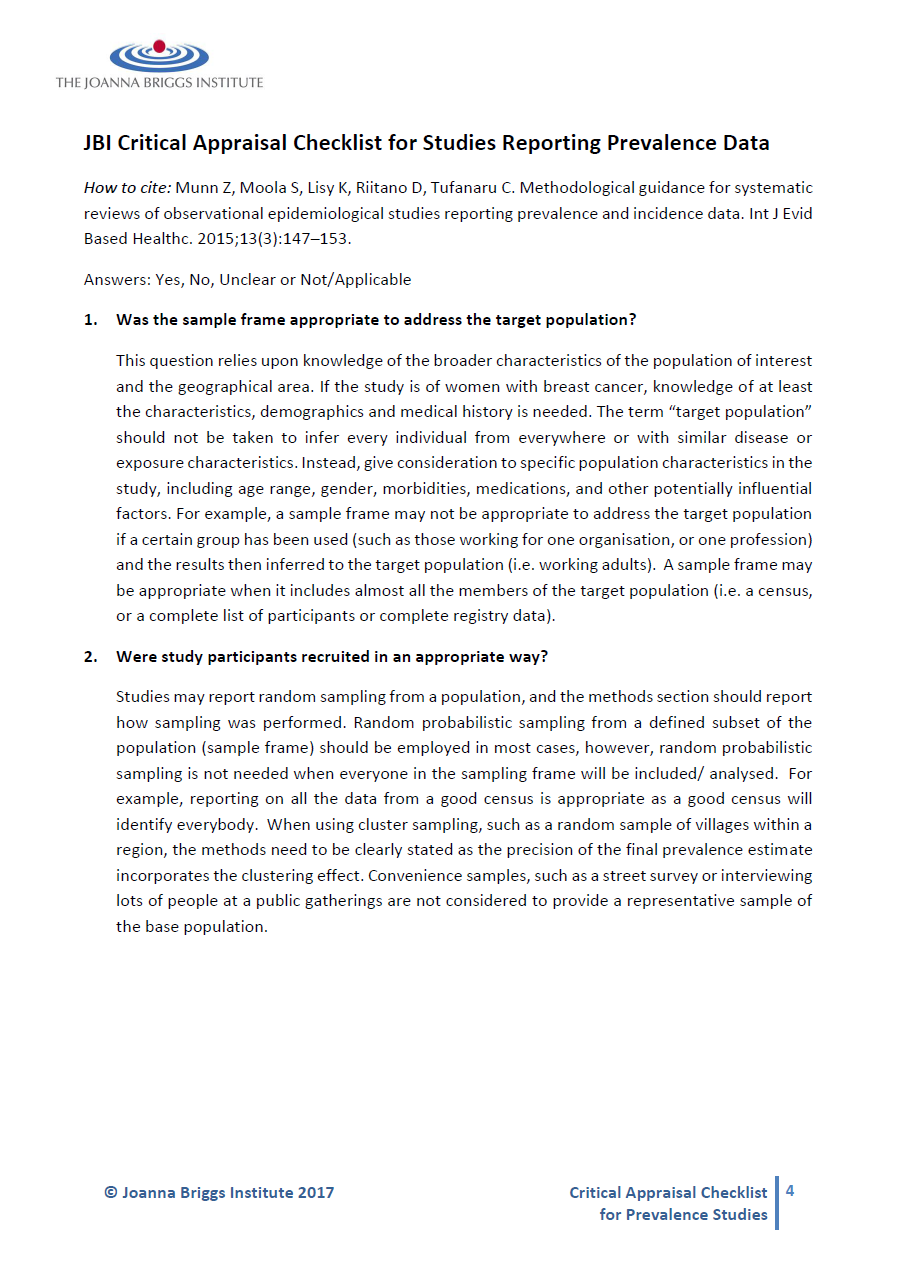


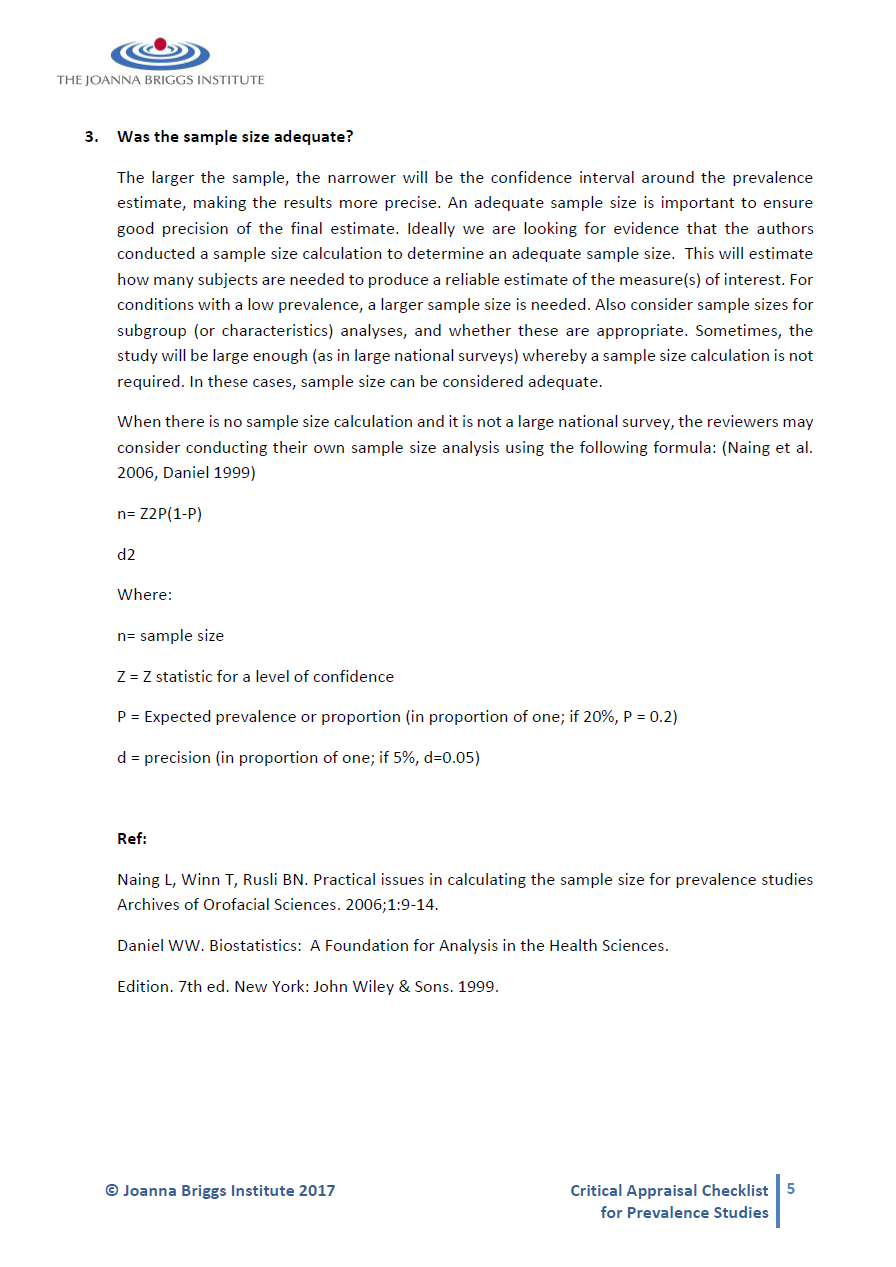


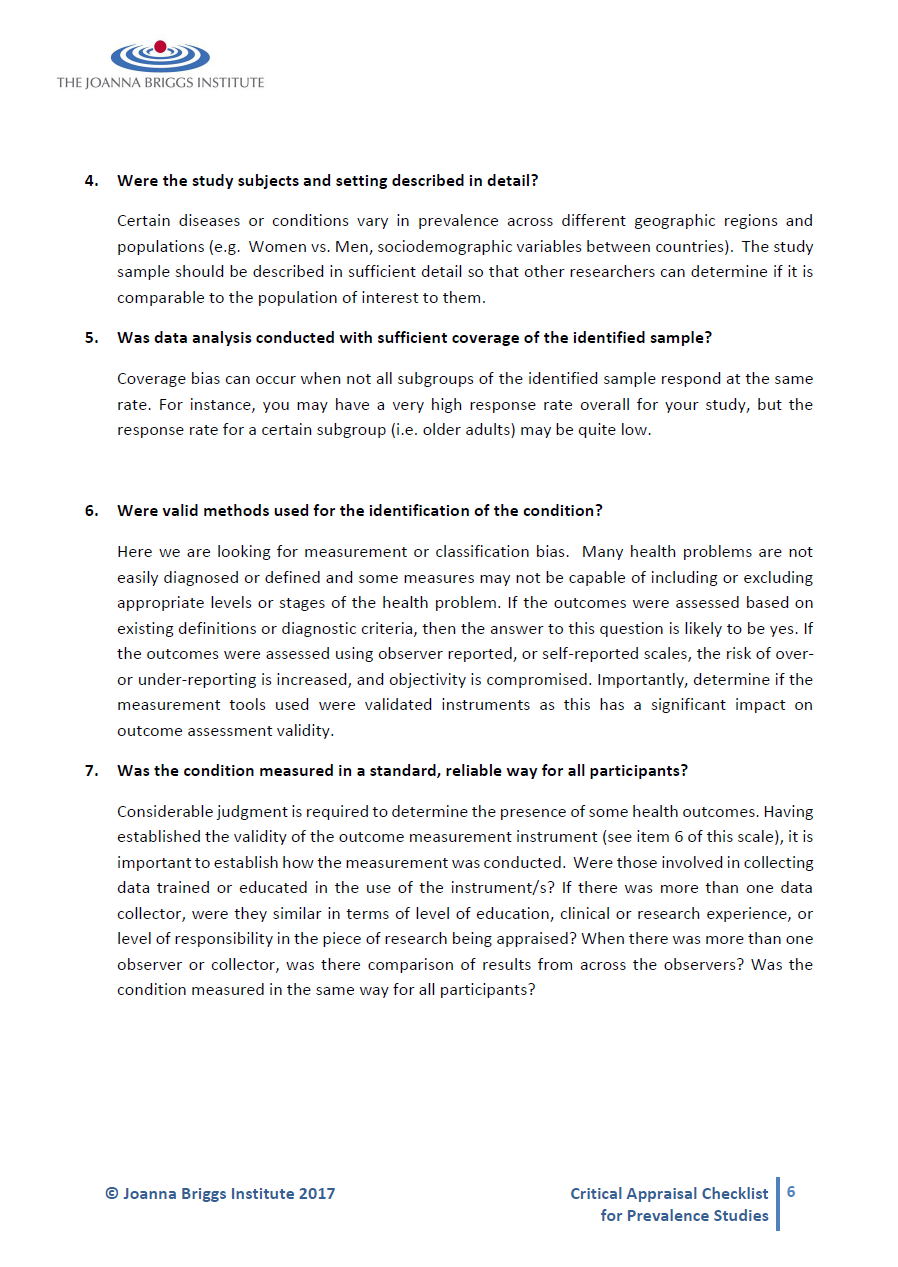


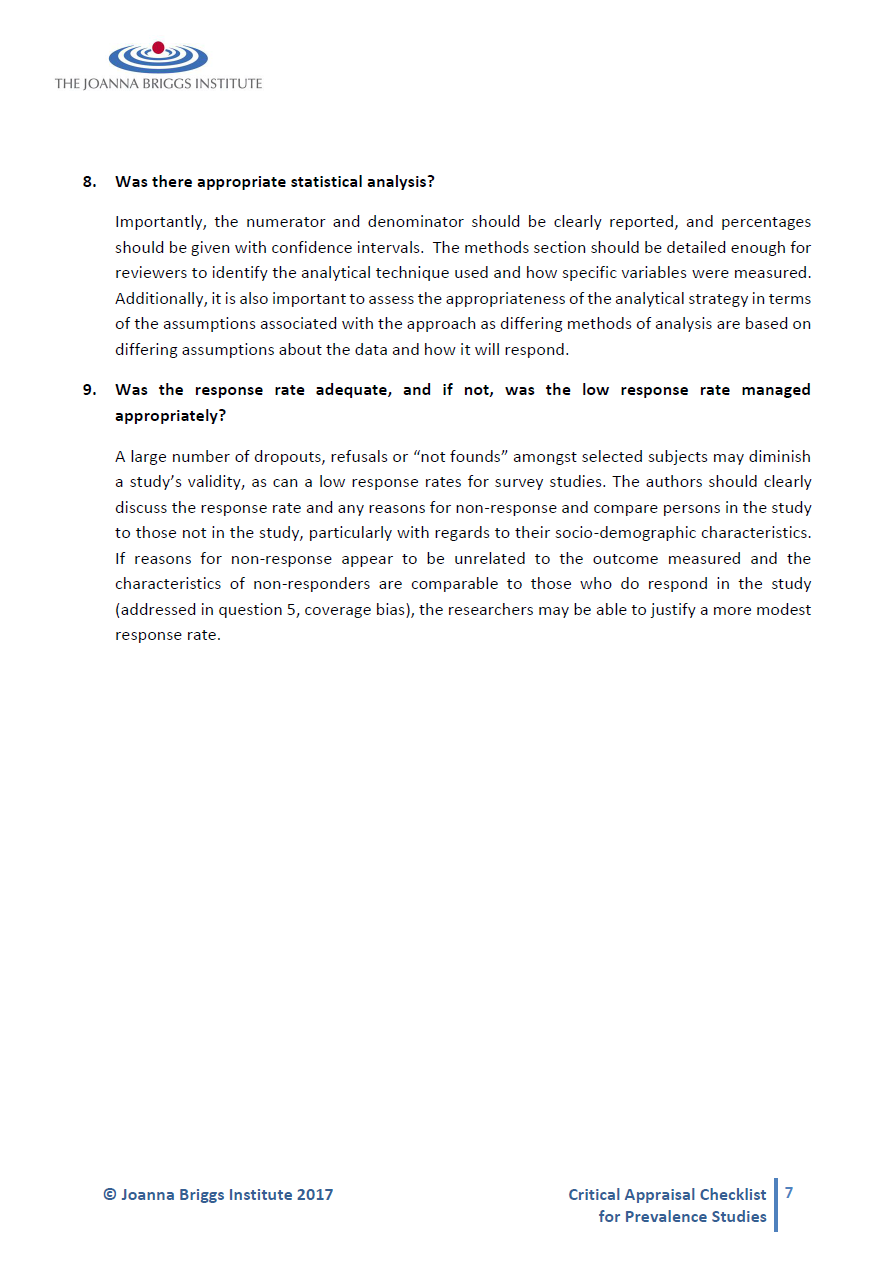


**Supplemental Methods 3:** Newcastle-Ottawa Risk of Bias Assessment Tool

**NEWCASTLE - OTTAWA QUALITY ASSESSMENT SCALE**

**COHORT STUDIES**

Note: A study can be awarded a maximum of one star for each numbered item within the Selection and Outcome categories. A maximum of two stars can be given for Comparability

**Selection**

1) Representativeness of the exposed cohort

a) truly representative of the average _______________ (describe) in the community **🟑**

b) somewhat representative of the average ______________ in the community **🟑**

c) selected group of users eg nurses, volunteers

d) no description of the derivation of the cohort

2) Selection of the non exposed cohort

a) drawn from the same community as the exposed cohort **🟑**

b) drawn from a different source

c) no description of the derivation of the non exposed cohort

3) Ascertainment of exposure

a) secure record (eg surgical records) **🟑**

b) structured interview **🟑**

c) written self report

d) no description

4) Demonstration that outcome of interest was not present at start of study

a) yes **🟑**

b) no

**Comparability**

1) Comparability of cohorts on the basis of the design or analysis

a) study controls for _____________ (select the most important factor) **🟑**

b) study controls for any additional factor **🟑** (This criteria could be modified to indicate specific control for a second important factor.)

**Outcome**

1) Assessment of outcome

a) independent blind assessment **🟑**

b) record linkage **🟑**

c) self report

d) no description

2) Was follow-up long enough for outcomes to occur

a) yes (select an adequate follow up period for outcome of interest) **🟑**

b) no

3) Adequacy of follow up of cohorts

a) complete follow up - all subjects accounted for **🟑**

b) subjects lost to follow up unlikely to introduce bias - small number lost - > ____ % (select an adequate %) follow up, or description provided of those lost) **🟑**

c) follow up rate < ____% (select an adequate %) and no description of those lost

d) no statement

**CODING MANUAL FOR COHORT STUDIES**

# SELECTION

1. **Representativeness of the Exposed Cohort**

Item is assessing the representativeness of exposed individuals in the community, not the representativeness of the sample of women from some general population. For example, subjects derived from groups likely to contain middle class, better educated, health oriented women are likely to be representative of postmenopausal estrogen users while they are not representative of all women (e.g. members of a health maintenance organisation (HMO) will be a representative sample of estrogen users. While the HMO may have an under-representation of ethnic groups, the poor, and poorly educated, these excluded groups are not the predominant users users of estrogen).

Allocation of stars as per rating sheet

1. **Selection of the Non-Exposed Cohort**

Allocation of stars as per rating sheet

1. **Ascertainment of Exposure**

Allocation of stars as per rating sheet

1. **Demonstration That Outcome of Interest Was Not Present at Start of Study**

In the case of mortality studies, outcome of interest is still the presence of a disease/ incident, rather than death. That is to say that a statement of no history of disease or incident earns a star.

***COMPARABILITY***

1. **Comparability of Cohorts on the Basis of the Design or Analysis**

A maximum of 2 stars can be allotted in this category

Either exposed and non-exposed individuals must be matched in the design and/or confounders must be adjusted for in the analysis. Statements of no differences between groups or that differences were not statistically significant are not sufficient for establishing comparability. Note: If the relative risk for the exposure of interest is adjusted for the confounders listed, then the groups will be considered to be comparable on each variable used in the adjustment.

There may be multiple ratings for this item for different categories of exposure (e.g. ever vs. never, current vs. previous or never)

Age = , Other controlled factors =

***OUTCOME***

1. **Assessment of Outcome**

For some outcomes (e.g. fractured hip), reference to the medical record is sufficient to satisfy the requirement for confirmation of the fracture. This would not be adequate for vertebral fracture outcomes where reference to x-rays would be required.

1. Independent or blind assessment stated in the paper, or confirmation of the outcome by reference to secure records (x-rays, medical records, etc.)
2. Record linkage (e.g. identified through ICD codes on database records)
3. Self-report (i.e. no reference to original medical records or x-rays to confirm the outcome)
4. No description.
5. **Was Follow-Up Long Enough for Outcomes to Occur**

An acceptable length of time should be decided before quality assessment begins (e.g. 5 yrs. for exposure to breast implants)

1. **Adequacy of Follow Up of Cohorts**

This item assesses the follow-up of the exposed and non-exposed cohorts to ensure that losses are not related to either the exposure or the outcome.

Allocation of stars as per rating sheet
